# Ethnobotanical study of wild edible plants in Karamara forest patches, Eastern Ethiopia

**DOI:** 10.1186/s41182-025-00851-0

**Published:** 2025-11-10

**Authors:** Getu Alemayehu, Ashebir Awoke, Zewdie Kassa

**Affiliations:** 1 Department of Biology, Jigjiga University, P.O.Box 1020, Jigjiga, Ethiopia; 2 Department of Biology, Mizan-Tepi University, P.O. Box 121, Tepi, Ethiopia; 3 Department of Biology, Salale University, P.O. Box 245, Fitche, Ethiopia

**Keywords:** Ethiopia, Ethnobotany, Food security, Indigenous knowledge, Karamara forest, Wild edible plants

## Abstract

**Background:**

Wild edible plants (WEPs) play a vital role in ensuring food security, enhancing nutrition, and preserving cultural heritage, particularly in dryland ecosystems. In Eastern Ethiopia, the Karamara forest patches host a rich diversity of WEPs; however, comprehensive ethnobotanical documentation remains scarce. This study aimed to document the diversity, utilization patterns, seasonal availability, and indigenous knowledge of WEPs, as well as to assess associated threats and conservation practices.

**Methods:**

A cross-sectional ethnobotanical survey was conducted from February 2023 to January 2024 involving 64 informants selected through purposive and snowball sampling techniques. Data were collected via semi-structured interviews, focus group discussions, market surveys, and guided field walks. Voucher specimens were collected and identified using the *Flora of Ethiopia and Eritrea* and verified with digital plant databases such as the International Plant Names Index (IPNI) and Plants of the World Online (POWO). Quantitative analyses included Relative Frequency of Citation (RFC), Preference and Direct Matrix Ranking. Statistical analyses (t-tests, ANOVA, and Pearson correlation) were used to examine variations in ethnobotanical knowledge across gender, age, literacy, and experience.

**Results:**

A total of 42 WEP species, belonging to 32 genera and 24 families, were documented, with shrubs (50%) and trees (33.3%) as the dominant growth forms. Fruits (69%) and leaves (14%) were the most commonly consumed parts. *Amaranthus caudatus* L., *Ficus sycomorus* L., and *Ziziphus mucronata* Willd. were the most preferred species. Ethnobotanical knowledge showed significant variation among informant groups (P < 0.05). Seasonal availability, collection methods, and marketability patterns reflected local adaptive strategies. Major threats identified included firewood collection, charcoal production, overgrazing, and drought. Community-based management and integration of WEPs into home gardens were recognized as promising conservation approaches.

**Conclusion:**

WEPs in the Karamara forest patches play a vital role in supporting dietary diversity, livelihoods, and cultural identity. Sustainable management, participatory conservation, and systematic documentation of indigenous knowledge are essential for safeguarding both biodiversity and food security in the region. Future studies should prioritize investigating the nutritional composition, phytochemical properties, and pharmacological potential of WEPs to ensure their safe utilization and explore broader applications.

## Background

In many developing countries, including Ethiopia, wild and semi-wild edible plants are central to rural diets. Hundreds of species are consumed as vegetables, fruits, or tubers, often supplementing cultivated staples and enriching grain-based diets with flavor, variety, vitamins, and minerals [1, 2]. In Ethiopia, WEPs are valued as supplementary, seasonal, or emergency foods across cultural groups, thereby contributing significantly to food security [3]. Despite their wide distribution, knowledge of their genetic diversity, nutritional value, and patterns of use remains limited [4, 5]. While food security research has largely focused on cultivated crops, increasing evidence shows that wild foods are indispensable to both global and local food systems, particularly forest species that provide essential macro- and micronutrients [6, 7].

Traditional knowledge of wild food plants, however, is under threat due to shifting values, land-use changes, and environmental degradation [2–4]. Erosion of this knowledge is especially evident among younger generations and urban communities, raising concern over the loss of ethnobotanical heritage that has sustained rural livelihoods for centuries [4, 5]. Documenting and preserving such knowledge is therefore crucial, both for safeguarding cultural traditions and for maintaining future options in food security and health interventions.

WEPs, defined as non-cultivated species gathered from forests, rangelands, and agro-ecological mosaics, provide benefits far beyond subsistence [6–9]. They diversify diets, serve as emergency foods during climatic and economic shocks, and carry cultural significance in local cuisines. Moreover, they support livelihoods through medicinal uses, fodder supply, and household economies [10–12]. Recent assessments highlight that food insecurity in sub-Saharan Africa is rising due to climate variability, conflict, and economic stress, making WEPs indispensable safety nets [8, 9]. Thus, documenting and sustaining WEPs is both an ethnobiological priority and a public health necessity.

Ethiopia is a hotspot of biodiversity and traditional knowledge, with more than 650 WEP species documented across 94 families [4]. These plants provide diverse edible parts including fruits, leaves, seeds, tubers, and shoots with fruits being most commonly consumed. Beyond their nutritional role, WEPs possess bioactive compounds with antioxidant and antimicrobial properties that help prevent micronutrient deficiencies and diet-related diseases [10, 11]. Despite these benefits, their integration into agricultural, health, and food policies remains limited. Localized studies have highlighted their market potential and seasonal availability, but major research gaps persist, particularly in underrepresented regions such as Eastern Ethiopia [2, 12–15].

The Karamara forest patches, located near Jijiga in the Upper Fafan catchment of the Somali Region, represent ecologically unique biodiversity refugia within predominantly pastoral and agropastoral landscapes. These patches are vital sources of fruits, leafy greens, and medicinal plants that support community food security and health. However, they are increasingly threatened by deforestation, overgrazing, recurrent droughts, and biological invasions, particularly by *Prosopis juliflora*, which displaces native vegetation and restricts access to foraging sites [16–18]. These pressures not only endanger the persistence of WEPs but also erode intergenerational knowledge of their use.

The conservation and sustainable management of WEPs align closely with several United Nations Sustainable Development Goals (SDGs). They contribute to SDG 2 (Zero Hunger) by enhancing dietary diversity and bridging seasonal food shortages; to SDG 3 (Good Health and Well-being) through their nutritional and medicinal properties; to SDG 12 (Responsible Consumption and Production) by promoting sustainable harvesting and value addition; and to SDG 15 (Life on Land) by supporting biodiversity conservation and ecosystem resilience. Documenting and analyzing WEPs in the Karamara forest patches is therefore vital for strengthening local food systems while advancing global sustainability agendas. This study aims to: (i) Document WEPs in Karamara forest patches of Eastern Ethiopia, including vernacular names, edible parts, preparation methods, and seasonal availability; (ii) assess their cultural significance using ethnobotanical indices; (iii) examine socio-demographic variations in knowledge and use across gender, age, and livelihood systems; and (v) identify ecological and anthropogenic threats while proposing conservation-oriented strategies aligned with the SDGs.

These objectives are guided by four hypotheses: (H₁) Karamara forest patches of Eastern Ethiopia hosts a diverse assemblage of WEPs actively integrated into local diets and cultural practices; (H₂) high-use species provide substantial nutritional contributions, particularly during lean seasons; (H₃) traditional knowledge varies significantly across socio-demographic groups, with older adults and women retaining greater expertise; and (H₄) key WEPs face mounting pressures from environmental change and human exploitation, requiring urgent conservation interventions. In addressing these aims, this study fills a critical ethnobotanical gap in the the study area and generates insights of direct relevance to food security, biodiversity conservation, and community well-being in Ethiopia.

## Materials and methods

### Description of the study area

Jijiga (Somali: Jigjiga) is a district within the Somali Region of Ethiopia, located in the Fafan Zone. The zone includes several towns and cities, such as Awbare, Derwernache, Lefe Isa, Babile, Kebri Beyah, Harshin, Goljano, Tuli Gulled, and Hart Sheik. Fafan Zone is bordered to the south by Jarar, to the southwest by Nogob, to the west by the Oromia Region, to the north by Sitti, and to the east by Somaliland. Jijiga district itself is bordered to the south by Kebri Beyah, to the southwest by Gursum, to the southeast by Ajersagora, to the northwest by Sitti Zone, and to the north by Awbare, with Jijiga town serving as the district capital.

The study was conducted in near Hadew project site, a sub-administrative unit within Jijiga district. The Karamara forest patches and Hadew project site are located at 9° 21′ – 9° 23′ North latitude and 42° 42′ – 42° 43′ East longitude, at an altitude ranging from 1921 to 2255 m above sea level. The site lies approximately 11 km west of Jijiga town and 13 km east of Fafan town. The study area comprises eight clusters: Halays, Ellebahay, Dudehidi, Hadew, Qordehaere, Kebelle 08, Jame’o, Haji Omer, Towfik, and Tawakal. The average elevation of the woreda is 1,803 m above sea level. The only perennial rivers in the area are the Fafen and Jerer.

Infrastructure in Jijiga includes 80 km of asphalt roads and 60 km of all-weather gravel roads, with approximately 34.1% of the population having access to drinking water. The Karamara mountains west of Jijiga were heavily mined during the Ogaden War, and some areas remain restricted due to unexploded ordnance.

Historically, before the 2004 October referendum that defined the disputed boundary between Oromia and Somali Regions, the northern section of this district became Chinaksen district, transferred to the Oromia Region. According to the 2007 Census by the Central Statistical Agency (CSA), Jijiga district had a population of 277,560, comprising 149,292 men and 128,268 women. Urban inhabitants accounted for 45.35% (125,876), while 2.51% (6,956) were pastoralists. The majority (91.41%) of the population are Muslim, with 6.97% practicing Orthodox Christianity. The woreda is predominantly inhabited by Somali subclans, including Gadabuursi, Ogaden, Abaskuul, Bartire, and Yabare of the Jidwaq; Geri Koombe; Habr Awal and Garhajis of the Isaaq; and Sheekhaal.

A 2001 sample enumeration by CSA of 37,413 farmers indicated an average landholding of 1.05 hectares per household. Of the 39.37 km^2^ of surveyed private land, 81.21% was cultivated, 10.6% used as pasture, 3.72% left fallow, and 1.8% allocated for other uses. Cropland was dominated by cereals (66.08%), with minor shares of pulses (1.61%), root crops (1.61%), and vegetables (0.07%). Permanent crops included 4,108 hectares of khat, one hectare of enset, and 14.55 hectares of fruit trees. About 78.33% of farmers practiced mixed crop-livestock production, 19.88% cultivated crops only, and 1.79% raised livestock exclusively. Land tenure was primarily owned (94.28%), with 1.29% rented and 4.43% under other forms of tenure.

### Reconnaissance survey and research design

A reconnaissance survey was carried out from February 12 to 29, 2023, to collect baseline information and guide the selection of study sites within the Karamara forest patches. During this phase, general information about the district and the forest patches was gathered. Preliminary data were obtained from the Jigjiga District Office of Agriculture and supplemented through consultations with local elders, kebele (village) leaders, forest guards, and other knowledgeable community members. This stage enabled the researchers to become acquainted with the area’s topography, land use patterns, vegetation cover, and accessibility. The study adopted a cross-sectional research design, integrating both qualitative and quantitative ethnobotanical methods, to systematically document WEPs and the indigenous knowledge associated with them in the selected communities of the district.

### Informant and site selection

Baseline data were first obtained from the Jigjiga District Office of Agriculture. Additional consultations with local elders, forest guards, and kebele (local administrative unit) leaders helped guide the selection of study sites. The study area was purposively chosen for its relatively rich vegetation, the community’s strong reliance on WEPs, and the absence of formal conservation initiatives addressing these resources.

A total of 64 informants (40 men and 24 women), aged between 15 and 80 years, participated in the study. Of these, 40 general informants were identified through a snowball sampling technique, targeting long-term residents with extensive indigenous knowledge of WEPs. These individuals were recognized by their communities as reliable sources of ethnobotanical information. Additionally, 24 key informants were purposively selected based on recommendations from community leaders and local experts. Key informants included traditional gatherers, vendors, cooks, elders, and other individuals possessing specialized knowledge of WEPs.

Informants were categorized into three age groups: young adults (15–30 years, 18.7%), middle-aged adults (31–49 years, 29.7%), and elders (50–80 years, 51.6%), following age classifications commonly applied in ethnobotanical studies. The sample consisted of 62.5% men (n = 40) and 37.5% women (n = 24), reflecting local gender roles in the collection, preparation, and utilization of WEPs. Regarding marital status, 62.5% were married, 28.1% single, and 9.4% divorced.

Educational backgrounds varied, with 70.3% of informants being illiterate (n = 45) and 29.7% literate (n = 19). Livelihood activities were diverse: 31.4% were engaged in livestock keeping, 38.6% in farming, 18.3% in trade, and 11.7% in charcoal production or firewood selling, while the remaining informants pursued other occupations (Table 1). This sampling approach ensured representation of both general community knowledge and specialized expertise, providing a comprehensive understanding of the use, management, and cultural significance of WEPs in the study area.

**Table 1:** Summary of study sites and sociodemographic characteristics of informants

Name of study sites	Altitude	GPS coordinates		Gender		Ethnicity (Somali)	Age categories			Language (Af Soomaali)	Ocu	Education level		NH	AE
		Latitude (N,S)	Longitude (E,W)	M	F		15–30	31–49	50–80			Illi	Lit		
Karamara forest patches	1754-2255 m	9°21′18"N	42°42′14"E	40	24	Somali	12	19	33	Af Soomaali	LH,FA,MC,CS,FW,HW	45	19	22,567	High-land
Total				64			64					64		22,567	

Illi = illiterate, Lit = literate, M = Male, F = Female, AE = Agro-ecology, NH = Number of households, Ocu = Occupation LH = livestock holders,  = farmers,MC = merchants,CS = Charcoal sellers,FW = fire wood sellers, HW = HW = House wife

### Vegetation sampling and ethnobotanical data collection

A systematic sampling design was adopted to achieve representative coverage of the forest vegetation. The study area was stratified based on altitude and disturbance gradients, with five transects established across each stratum. Along each transect, fifty main plots measuring 20 m × 20 m were laid at 100 m intervals to record woody wild edible plant species. Within each main plot, subplots measuring 5 m × 5 m were nested for sampling shrubs and herbs.

### Semi-structured interviews

Ethnobotanical data were collected from February 2023 to January 2024 using standard ethnobotanical methodologies [27, 28]. Semi-structured interviews were employed to obtain detailed information on WEPs. A set of guiding questions was prepared to cover key topics, including local plant names, growth habits, collection methods, edible parts, preparation and consumption methods, seasonal availability, perceived nutritional values, threats, and management practices.

The interviews were conducted in Somali (Af-Soomaali), the local language, with the assistance of experienced local translators, and responses were recorded in English. Each interview was held individually with informants who were either native to the area or had lived there most of their lives, ensuring the reliability and authenticity of local knowledge. Conducting one-on-one interviews helped minimize group influence and ensured the accuracy of individual responses.

Permission for WEP collection and data gathering was obtained from landowners, as well as from the district administrative and agricultural offices, in accordance with established ethical research guidelines.

### Guided field walks and observations

Guided field walks, also referred to as “walks in the forest patches,” were conducted with key informants to document WEPs in their natural habitats. During these walks, local informants accompanied researchers to identify and locate plant species within the forest. Morphological features, growth habits, and ecological conditions were carefully recorded in situ.

Voucher specimens were collected, placed in labeled polyethylene bags, and later pressed, air-dried, or deep-frozen for proper preservation and identification. Photographic documentation was also undertaken to complement field observations and ensure accurate species verification.

In addition to specimen collection, detailed ethnobotanical information was gathered during these walks, including vernacular names, edible parts, preparation methods, and other traditional uses of each species. Observations were also made on the main threats to WEPs, local conservation practices, and the transmission of indigenous knowledge across generations.

### Focus group discussions

Focus group discussions (FGDs) were held to document indigenous knowledge and practices associated with nutritionally important WEPs. Four FGDs were conducted, each comprising 6–8 participants (aged 20–75 years) of mixed gender. Participants were purposively selected based on their experience and recognition within the community for their knowledge of local plant use.

The discussions encouraged open and interactive dialogue, facilitating the exchange of diverse views and experiences. Topics explored included locally consumed species, vernacular plant names, edible parts, methods of preparation and consumption, seasonal availability, and local practices related to the management and conservation of WEPs and surrounding vegetation.

### Wild edible plant specimen collection and identification

Voucher specimens were collected with the assistance of informants and local field guides. Field notes and photographs were taken to document associated indigenous knowledge and the ecological settings of each species. The collected specimens were transported to the Biology Department Laboratory, College of Natural and Computational Sciences, Jigjiga University, where they were air-dried, pressed, labeled, numbered, and preliminarily identified to the family or, when possible, species level.

Further taxonomic identification was carried out using the Flora of Ethiopia and Eritrea and standard botanical identification keys. Verification was conducted at the National Herbarium (ETH) of Addis Ababa University by comparing the collected specimens with authenticated herbarium materials and relevant taxonomic references [19, 20]. Scientific nomenclature followed the accepted standards in the Flora of Ethiopia and Eritrea.

Botanical names were cross-checked and updated using the International Plant Names Index (IPNI) and Plants of the World Online (POWO). Additional digital resources, including PlantNet, Flora Finder, PlantSnap, Google Image Search, the African Plant Database, the World Checklist of Selected Plant Families, and JSTOR Global Plants [2, 21, 22], were consulted to validate nomenclature and classification. This integrated approach ensured accurate identification and reliable documentation of WEPs in the Karamara forest patches.

### Quantitative ethnobotanical analysis

To complement qualitative findings, several quantitative ethnobotanical tools were applied to assess the cultural significance, knowledge distribution, and conservation status of WEPs in the study area.

### Botanical ethnoknowledge index (BEI)

The Botanical Ethnoknowledge Index (BEI) was employed to quantify the extent and distribution of ethnobotanical knowledge within the study population and to allow comparisons across groups inhabiting similar ecological and floristic settings [23]. The BEI integrates multiple variables, including: (i) the total number of plant species reported by all members of a group, (ii) the mean number of species cited per participant, (iii) the mean number of citations per species, (iv) the number of participants in each group, and (v) the total number of species reported across all groups in the study.

The index produces values ranging from just above 0 to a theoretical maximum of 2. Higher BEI values reflect a greater depth and breadth of ethnobotanical knowledge within the group. While values approaching 2 are possible, they are rare and generally indicate distinct or highly specialized knowledge relative to other groups.

The BEI is calculated using the following formula:

$${\text{BEI}} = (\frac{{{\text{ms}}}}{{{\text{sg}}}}\, + \,\frac{{{\text{ms}}}}{{\text{N}}}) \times \frac{{{\text{sg}}}}{{{\text{st}}}}$$

where, BEI: Botanical Ethnoknowledge Index. ms: mean number of species reported per participant in a particular group. Sg: total number of species reported by all participants in a particular group. mc: mean number of citations per species in a particular group. N: number of participants in the particular group. St: total number of species reported by all compared groups in the study. The above-presented index is designed to compare groups with similar sample sizes [23]. However, in order to overcome this limitation when comparing groups with diferent sample sizes, the index value can be relatively corrected by multiplying it with a coefcient as follows:

$$\text{F}=\frac{N mean}{N mean+ \sqrt{N}mean}$$

where, F: correcting factor. N mean: mean number of participants among all compared groups. √N min: square root of the number of participants in the smallest group.

### Relative frequency of citation (RFC)

The Relative Frequency of Citation (RFC) was used to assess the local importance of each species based on how frequently it was mentioned by informants. RFC values range from 0 (no mention) to 1 (maximum mention), with higher values reflecting greater cultural salience and reliance on a particular species [26]. The RFC was calculated as:

$${\text{RFC}} = \frac{{{\text{FC}}}}{{\text{N}}}$$

where; FC = the number of informants citing the use of the species and N = the total number of respondents participating in the survey.

### Preference ranking and quantitative assessments

#### Preference ranking

Preference ranking of WEPs was conducted following established ethnobotanical methods [27, 28]. Informants evaluated the most frequently cited WEPs based on taste, availability, accessibility, cultural significance, and income-generating potential. Each species was scored on a scale from 1 (least preferred) to 5 (most preferred). Species with the sweetest taste or highest desirability received a score of 5, while the least preferred species received a score of 1. Scores from all informants were summed to determine the overall rank of each species, providing insight into the culturally and nutritionally most valued WEPs within the study communities.

#### Direct matrix ranking (DMR)

To assess the multifunctional roles of WEPs beyond their dietary use, direct matrix ranking was applied to six multipurpose species [27, 28]. Key informants assigned scores from 0 (no use) to 5 (highest use) across seven categories: food, medicine, fodder, firewood, charcoal production, construction, and income generation. Average scores for each category were calculated, and cumulative scores were used to rank species according to their multifunctional importance. This method identified species most heavily relied upon by local communities and those under the greatest utilization pressure.

#### Priority ranking of threats

Priority ranking was conducted with key informants to identify the main threats affecting WEPs. Seven major threats were assessed: agricultural expansion, charcoal production, introduction of exotic species, firewood collection, construction and tool use, overgrazing, and persistent drought. Informants scored each threat on a scale from 1 (least severe) to 6 (most severe). Scores were summed across informants to produce a composite ranking, highlighting the most critical ecological, economic, and cultural pressures driving WEP decline in the study area.

#### Data analysis

Field data were systematically organized, including local and scientific names, botanical families, life forms, edible parts, and habitats. Records were maintained in Microsoft Word 2019, and descriptive statistics were used to summarize the data. Frequency distributions were presented using tables, pie charts, and bar graphs, and statistical analyses were performed in R software (version 4.4.2).

To assess variation in ethnobotanical knowledge across informant categories, the Botanical Ethnoknowledge Index (BEI) was applied. Data normality was verified using the Shapiro–Wilk test prior to parametric analyses. Independent t-tests were conducted to evaluate differences in WEP knowledge between genders, educational levels, and among participants with and without WEP experience. One-way ANOVA examined knowledge variation among age groups, while Pearson’s correlation analysis assessed the relationship between age and the number of WEPs reported [2].

#### Ethical considerations

Official permission for the study was obtained from the Department of Biology, Jigjiga University, and approved by the administrative offices of Jigjiga district. The research adhered to the Code of Ethics of the International Society of Ethnobiology (ISE). Prior informed consent was obtained from all participants after clearly explaining the study’s objectives, scope, and significance. Participants were informed that their involvement was voluntary, and that the research had no commercial intent, being conducted solely for academic and community benefit.

Cultural values and traditional knowledge systems were respected throughout the study. Informants were informed that their contributions would support the documentation and preservation of ethnobotanical knowledge, which is essential for local food security, cultural identity, and biodiversity conservation. The study emphasized promoting the sustainable use of WEPs for the well-being of both current and future generations.

## Results

### Diversity of wild edible plants in the Karamara forest patches

The ethnobotanical survey conducted in the Karamara forest patches recorded a total of 42 wild edible plant (WEP) species, belonging to 32 genera and 24 families (Table 2). Among these, Fabaceae was the most dominant family, with six species *Acacia mellifera, Acacia tortilis, Senegalia senegal, Tamarindus indica, Vachellia seyal,* and *Senna occidentalis*. Other well-represented families included Moraceae and Boraginaceae, each contributing three species. Notable species include *Ficus sycomorus, Ficus vasta,* and *Cordia africana*, recognized for their edible fruits, shade provision, and multifunctional roles in local livelihoods. Similarly, the Malvaceae family contributed three species (*Grewia mollis, Grewia schweinfurthii,* and *Grewia tenax*), whose fruits serve as important supplementary and famine foods.

**Table 2:** Wild edible plants list collected from Karamara forest patches

Family name	Scientific name	Local language	Pu	Ha	MoC	Mo.con	SP	RU	VN
Amaranthaceae	*Amaranthus caudatus* L.*	Cayo-geel, Milaxbuur	Se, L	H	Plucking	Cooked	Cf	AF	GA06
	*Amaranthus dubius* Mart. ex Thell.*	Cayo-geel, Milaxbuur	Se, L	H	Plucking	Cooked	Cf	AF	GA07
	*Amaranthus spinosus* L.*	Cayo-geel, Milaxbuur	Se, L	H	Plucking	Cooked	Cf	AF	GA08
Anacardiaceae	*Rhus vulgaris* Meikle	Dhadays, Kimo (Or)	Fr	Sh	Picking	Raw	Fo	AF	GA31
Apocynaceae	*Acokanthera schimperi* (A.DC.) Schweinf	Waab ay	Fr	Sh	Picking	Raw	Fo	AF	GA05
	*Carissa edulis* (Forssk.) Vahl	Agamse	Fr	Sh	Picking	Raw	Fo	Fam	GA12
Boraginaceae	*Cordia africana* Lam	Madheedh	Fr	T	Picking	Raw	Fo	Fam	GA14
	*Cordia sinensis* Lam	Madheedh	Fr	T	Picking	Raw	Fo	Fam	GA15
	*Ehretia cymosa* Thonn	Minegure	Fr	Sh	Picking	Raw	Fo	AF	GA19
Cactaceae	*Opuntia ficus-indica* (L.) Mill. *	Qoora	Fr	Sh	Picking	Raw	Cf	AF	GA29
Commelinaceae	*Commelina latifolia* Hochst. ex A.Rich	Baar	L	H	Plucking	Cooked	Fo	Fam	GA13
Cucurbitaceae	*Cucumis kelleri* (Cogn.) Ghebret. & Thulin	Uneexo	Fr	H	Picking	Raw	Fo	Fam	GA16
Ebenaceae	*Euclea racemosa* L	Miessa (Or)	Fr	Sh	Picking	Raw	Fo	Fam	GA20
Euphorbiaceae	*Ricinus communis* L	Tsema (Or)	Fr	Sh	Picking	Raw	Fo	AF	GA32
Fabaceae	*Acacia mellifera* (Vahl) Benth	Bilcil/Bilcin	Fr	T	Picking	Raw	Fo	AF	GA01
	*Acacia tortilis* (Forssk.) Hayne	Assel, Qudhac	Gu	T	Plucking	Raw	Fo	AF	GA04
	*Senegalia senegal* (L.) Britton *	Geedka Xabagta	Gu	T	Plucking	Raw	Cf	AF	GA02
	*Senna occidentalis* (L.) Link	Dabaysha	Fr	Sh	Picking	Raw	Fo	Fam	GA36
	*Tamarindus indica* L	Roqa	Fr	T	Picking	Raw	Fo	Fam	GA38
	*Vachellia seyal* (Delile) P.J.H.Hurter*	Shema (Or)	Gu	T	Plucking	Raw	Cf	AF	GA03
Malvaceae	*Grewia mollis* Juss	Gomosh	Fr	Sh	Picking	Raw	Fo	AF	GA23
	*Grewia schweinfurthii* Burret	Dhabi Dhoqosh	Fr	Sh	Picking	Raw	Fo	AF	GA24
	*Grewia tenax* (Forssk.) Fiori	Dhafaruur	Fr	Sh	Picking	Raw	Fo	AF	GA25
Moraceae	*Ficus sycomorus* L	Darey	Fr	T	Picking	Raw	Fo	AF	GA21
	*Ficus vasta* Forssk	Berda	Fr	T	Picking	Raw	Fo	AF	GA22
Moringaceae	*Moringa stenopetala* (Baker f.) Cufod. *	Moringa	L	T	Plucking	Cooked	Cf	AF	GA27
Olacaceae	*Ximenia afra* Sond	Hundhe	Fr	T	Picking	Raw	Fo	Fam	GA39
Polygonaceae	*Rumex nervosus* Vahl	Dhangaggoo(Or)	Wp	H	Plucking	Raw	Fo	Fam	GA35
Primulaceae	*Myrsine africana* L	Isburaan	Fr	Sh	Picking	Raw	Fo	AF	GA28
Rhamnaceae	*Ziziphus mucronata* Willd	Kasil/Eddi-shebel	Fr	Sh	Picking	Raw	Fo	AF	GA40
	*Ziziphus spina-christi* (L.) Desf.*	Kasil/Eddi-shebel	Fr	Sh	Picking	Raw	Cf	AF	GA42
	*Ziziphus mauritiana* Lam	Kasil/Eddi-shebel	Fr	Sh	Picking	Raw	Cf	AF	GA41
Rosaceae	*Rosa abyssinica* R.Br. ex Lindl	Goro (Or)	Fr	Sh	Picking	Raw	Fo	AF	GA33
	*Rubus volkensii* Engl	Dhangagoo (Or)	Wp	Sh	Plucking	Raw	Fo	Fam	GA34
Rubiaceae	*Canthium arboreum* S.Vidal	Qoodho orgi	Fr	Sh	Picking	Raw	Fo	AF	GA10
	*Canthium coromandelicum* (Burm.f.) Alston	Timiro	Fr	Sh	Picking	Raw	Cf	AF	GA11
Salicaceae	*Dovyalis abyssinica* (A.Rich.) Warb	Ongolatz	Fr	Sh	Picking	Raw	Fo	Fam	GA18
Santalaceae	*Osyris quadripartita* Salzm. ex Decne	Geed shimbereed	Fr	Sh	Picking	Raw	Fo	AF	GA30
Solanaceae	*Datura stramonium* L. *	Churuko	Fn	H	Plucking	Raw	Cf	Fam	GA17
	*Solanum nigrum* L. *	Adua (Or)	L	H	Plucking	Cooked	Hg	Fam	GA37
Verbenaceae	*Lantana camara* L.*	Hamarreessa (Or)	Fr	H	Picking	Raw	Cf	AF	GA26
Zygophyllaceae	*Balanites aegyptiaca* (L.) Delile*	Got, Gueza, Gut, Kulen	Fr	T	Picking	Raw	Fo	AF	GA09

Fr = fruit, Ro = root, S = stem, L = leaf, Fn = flower nectar, Gu = gum, Se = seed, Wp = whole plant, Hg = Homegarden, Method of collection = MoC, Fo = Forest, Cf = Cultiated filed, * Indicate the cultivated species,RU = Reason of use, Or = Oromiffa, Mo = Mode of consumption, Ln = Languge, Pu = Part used, Ha = habit, SP = Source of plant, AF = Additional food and Fam = Famine

Several families contributed two species each, such as Rhamnaceae (*Ziziphus mucronata, Ziziphus spina-christi*), Boraginaceae (*Cordia sinensis, Ehretia cymosa*), and Rosaceae (*Rosa abyssinica, Rubus volkensii*). Single-species families including Moringaceae (*Moringa stenopetala*), Olacaceae (*Ximenia afra*), Zygophyllaceae (*Balanites aegyptiaca*), and Cactaceae (*Opuntia ficus-indica*) highlight the specialized contributions of unique taxa to local food systems.

Of the 42 species recorded, 10 were identified as cultivated or semi-cultivated, including *Amaranthus spp., Moringa stenopetala, Senegalia senegal,* and *Opuntia ficus-indica*, while the majority were strictly wild-harvested. Culturally significant species, such as *Ficus sycomorus, Moringa stenopetala,* and *Carissa edulis*, illustrate the deep-rooted ethnobotanical knowledge of the Somali Region.

### Life forms of wild edible plant species

**Fig. 1:**
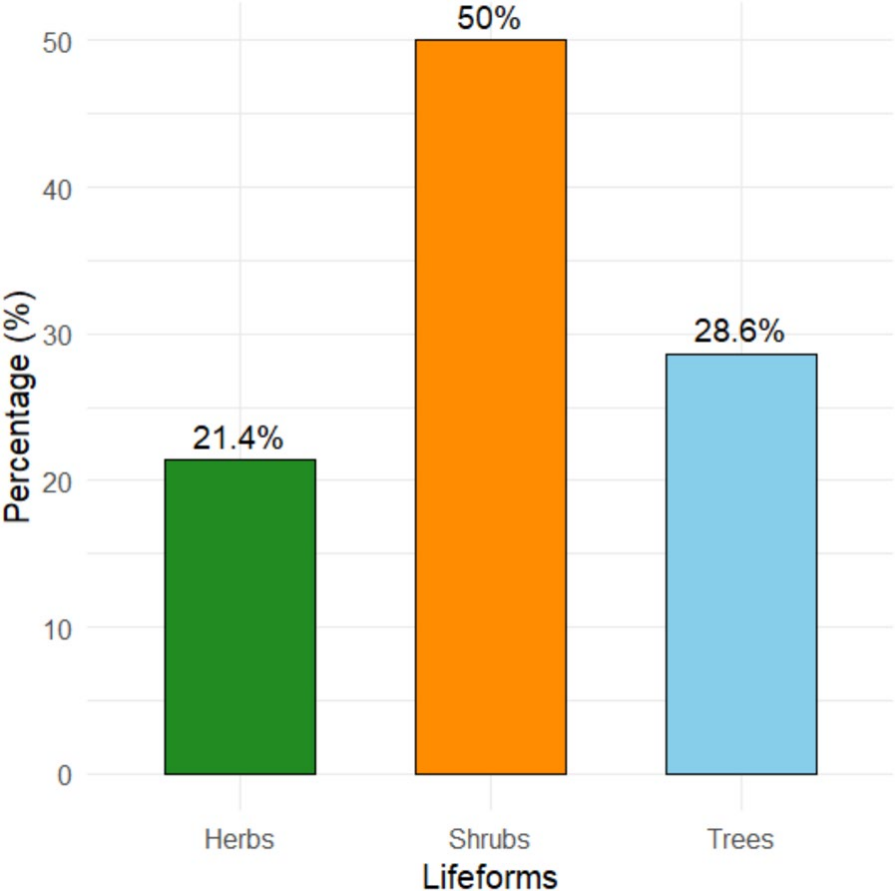
Distribution of lifeforms among wild edible plant species

Analysis of the life forms of WEPs documented in the Karamara forest patches revealed substantial diversity across different growth habits. Among the 42 species recorded, three major life forms were identified: trees (T), shrubs (Sh), and herbs (H) (Fig. 1).

Shrubs were the most dominant growth form, accounting for 21 species (50%) of the total. Notable examples include *Carissa edulis, Grewia spp., Ziziphus spp.,* and *Rosa abyssinica*.

Trees represented the second most abundant category, with 14 species (33.3%). Key species include *Cordia africana, Ficus sycomorus, Tamarindus indica, Moringa stenopetala,* and *Balanites aegyptiaca*. Herbs were the least represented growth form, comprising 7 species (16.7%), including *Amaranthus spp., Commelina latifolia, Rumex nervosus, Solanum nigrum,* and *Datura stramonium*. Despite their lower abundance, herbs are nutritionally significant, particularly as sources of leafy vegetables rich in vitamins and minerals. They are predominantly consumed during the rainy season, when fresh greens are abundant, making them important contributors to dietary diversity and seasonal food security.

### Edible parts and modes of consumption

**Fig. 2:**
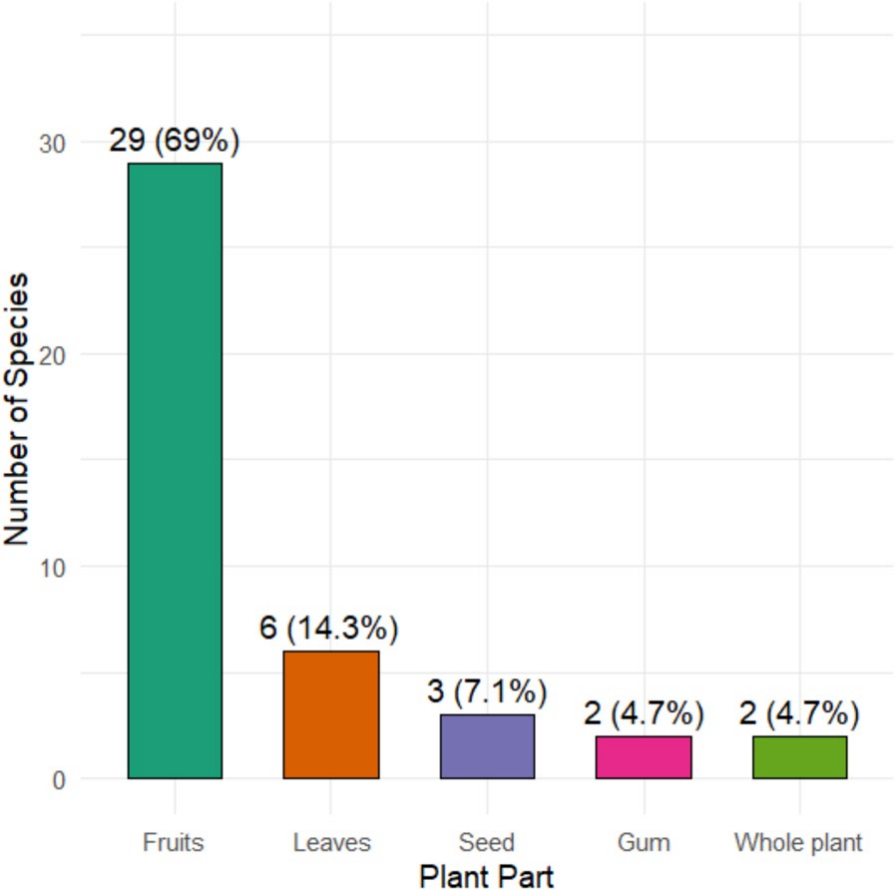
Edible parts of wild edible plant species

Analysis of the edible parts of WEPs documented in the Karamara forest patches revealed that local communities utilize a wide variety of plant structures for food. Across the 42 recorded species, six major edible categories were identified: fruits, leaves, seeds, gums, whole plants, and flower nectar (Fig. 2).

Fruits were the most commonly consumed part, recorded in 29 species (69%), including *Carissa edulis, Cordia africana, Grewia spp., Ziziphus spp., Tamarindus indica,* and *Balanites aegyptiaca*. Fruits were predominantly eaten raw, either fresh or occasionally dried for later use. Their palatability, seasonal availability, and portability make them primary contributors to local diets.

Leaves were the second most frequently consumed part, recorded in six species (14%), such as *Amaranthus spp., Moringa stenopetala, Commelina latifolia,* and *Solanum nigrum*. Leaves were generally cooked before consumption, often prepared as vegetables, sauces, or soups. Leafy WEPs are highly valued for their nutritional contributions, particularly vitamins and minerals, and are commonly consumed during the rainy season when fresh greens are abundant.

Seeds were reported in three Amaranthus species (7%), typically consumed cooked as porridge or mixed with cereals, reflecting the integration of semi-cultivated Amaranthus into local diets and highlighting their dual status as both wild and domesticated resources.

Gums were harvested from three leguminous trees (*Acacia tortilis, Senegalia senegal,* and *Vachellia seyal*, 7%). These exudates are eaten raw, often chewed as snacks, and are particularly important for children and herders during long periods in the field. Gums provide energy as well as cultural significance as traditional dryland foods.

Whole plants were consumed in two species (*Rumex nervosus* and *Rubus volkensii*, 5%), typically eaten raw as famine or supplementary foods, reflecting their role as coping strategies during periods of acute food scarcity.

Flower nectar was documented in one species (*Datura stramonium*), consumed raw. Although representing a minor proportion of WEP use.

Regarding modes of consumption, the majority of WEPs were eaten raw (34 species; 81%), largely associated with fruits and gums. Cooked preparations were reported for eight species (19%), primarily leafy vegetables and seeds.

### Collection methods of wild edible plants

**Fig. 3:**
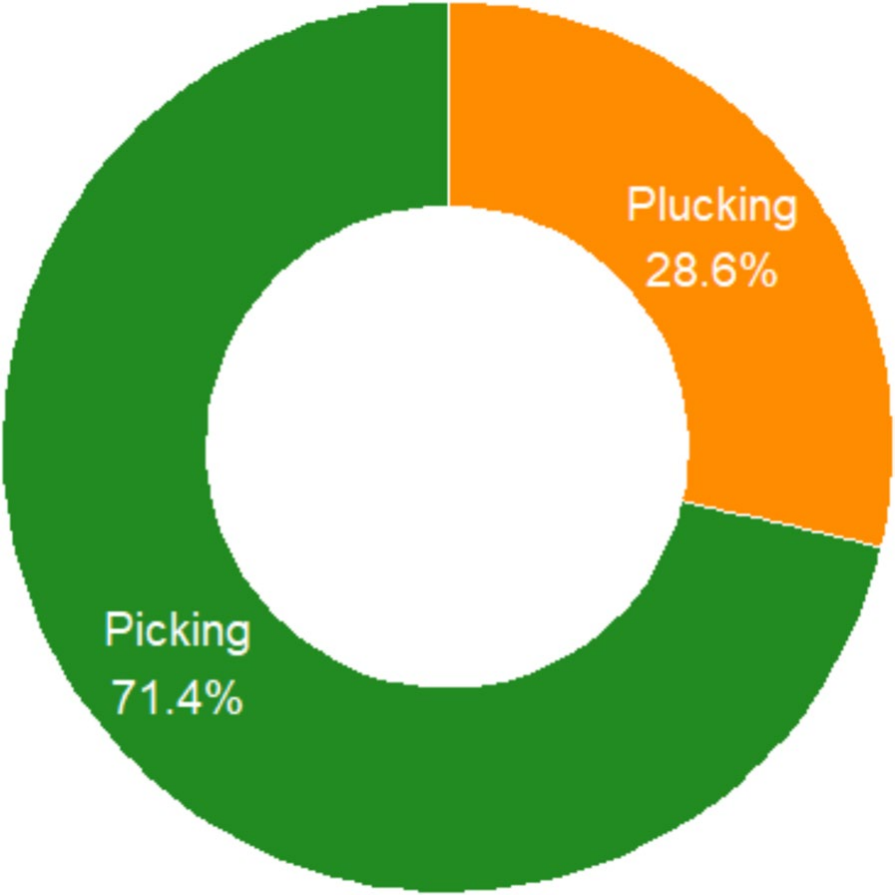
Collection methods of wild edible plants

The study of collection methods for WEPs in the Karamara forest patches revealed that local communities employ simple, traditional harvesting strategies tailored to the edible parts and growth habits of species. Two primary methods were identified: picking and plucking, each with distinct patterns of use (Fig. 3).

Picking was the most frequently employed method, applied to 30 species (71.4%), particularly fruit-bearing woody plants such as *Cordia africana, Ficus spp., Grewia spp.,* and *Ziziphus spp.*This technique involves hand-gathering ripe fruits directly from shrubs or trees, either by climbing, shaking branches, or collecting fallen fruits from the ground. Picking is a low-cost, minimally destructive method that supports sustainable harvesting over multiple fruiting periods. Plucking was reported in 12 species (28.6%), mainly associated with leafy vegetables (*Amaranthus spp., Solanum nigrum, Commelina latifolia, Moringa stenopetala*) and gums (*Acacia tortilis, Senegalia senegal, Vachellia seyal*). Plucking involves removing tender leaves, young shoots, seeds, or exudates by hand. Unlike picking, plucking can be labor-intensive and occasionally destructive, particularly when young leaves or stems are harvested repeatedly.

### Consumption period of wild edible plants

**Fig. 4:**
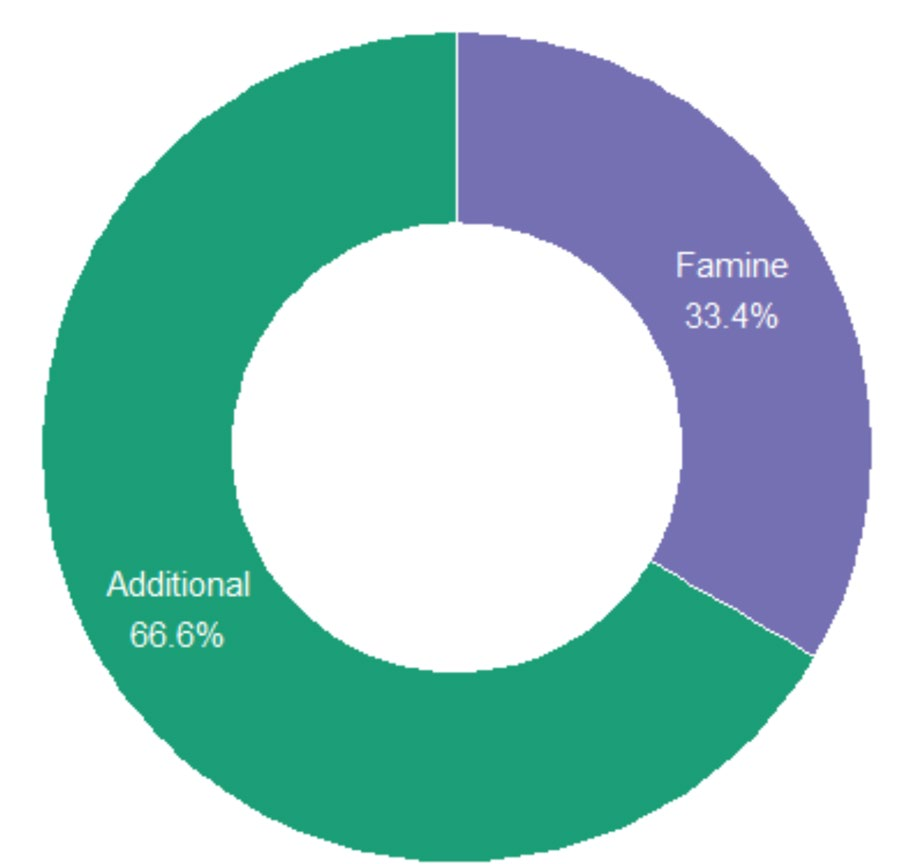
Consumption period of wild edible plants

The 42 recorded WEP species from the Karamara forest patches were classified based on their role in local diets: additional foods (AF) and famine foods (Fam) (Fig. 4). Additional foods (AF) accounted for 27 species (64.3%), including *Amaranthus spp., Opuntia ficus-indica, Acacia spp.,* and *Balanites aegyptiaca*. These species are incorporated into daily or seasonal diets, providing vitamins, minerals, and calories to complement staple grains. Many AF species, such as leafy vegetables (*Amaranthus spp., Moringa stenopetala*) and fruits (*Ficus sycomorus, Ziziphus mucronata*), are consumed during the rainy and post-rainy seasons.

Famine foods (Fam) comprised 15 species (35.7%), including *Cordia africana, Cucumis kelleri, Rumex nervosus, Datura stramonium,* and *Solanum nigrum*. These species are typically consumed during periods of acute food shortage, such as droughts or crop failures.

### Seasonal availability of wild edible plants

Among the 42 documented species, distinct seasonal patterns were observed: some species were available year-round, while others were restricted to specific periods following the rainy season.

Rainy-season species were predominantly herbaceous and leafy plants, including *Amaranthus caudatus, Commelina latifolia,* and *Solanum nigrum*.

Fruiting species, such as *Cordia africana, Ziziphus spp., Tamarindus indica,* and *Balanites aegyptiaca*, generally peak after the main rainy season.

Drought-tolerant and persistent species, including *Acacia mellifera, Acacia tortilis, Senegalia senegal,* and *Vachellia seyal*, remain accessible even during dry periods, providing gums, seeds, or leaves. These species act as emergency foods, serving as dietary buffers during lean seasons or drought years.

Famine and opportunistic species, such as *Rumex nervosus, Rubus volkensii, Dovyalis abyssinica,* and *Datura stramonium*, are harvested when other food resources are scarce.

### Marketability of wild edible plants

Among the 42 documented species, 12 species (28%) were reported as having market value, primarily those bearing edible fruits and seeds. Notable marketable species include *Amaranthus spp., Carissa edulis, Moringa stenopetala, Cordia africana, Ficus sycomorus, Grewia spp., Ziziphus spp., Tamarindus indica,* and *Balanites aegyptiaca*. These plants are harvested from forest patches, forest edges, and cultivated fields, then sold in local town markets or along trade routes connecting rural and urban centers.

Fruits dominated the marketable WEPs, reflecting high consumer demand and ease of storage and transport. Species such as *Ficus sycomorus, Dovyalis abyssinica, Grewia tenax,* and *Ziziphus mauritiana* are particularly valued for sweetness, nutritional content, and multi-month shelf life. Leafy vegetables, such as *Moringa stenopetala* and *Amaranthus spp.*, are marketed fresh during the rainy season, when supply is abundant.

Marketability is strongly influenced by species abundance, palatability, seasonality, and proximity to trade routes. Fruits and leaves from accessible forest edges or home gardens are more likely to reach markets than species from remote or degraded patches. Additionally, species culturally recognized as edible and safe tend to have higher market demand.

### Adverse effects associated with consumption of wild edible plants

WEPs in the Karamara forest patches contribute significantly to dietary diversity, nutrition, and household food security. While the majority of species are consumed safely, some carry potential adverse effects if used improperly, highlighting the importance of awareness and safe preparation practices.

Among the 42 documented species, most fruits, leafy vegetables, and seeds such as those from *Amaranthus spp., Ficus spp., Ziziphus spp., Grewia spp.,* and *Balanites aegyptiaca* are routinely incorporated into household diets without reported health issues.

However, certain species are recognized locally for their toxicity or health risks. *Datura stramonium L.* contains potent alkaloids and is considered toxic if consumed in large quantities or without proper preparation. Its use is generally limited to ritual or medicinal contexts, as accidental ingestion can cause hallucinations, gastrointestinal distress, or severe neurological effects. Similarly, *Solanum nigrum L.* is safe when cooked, particularly the young leaves and shoots, but raw ingestion or excessive consumption may lead to gastrointestinal irritation due to solanine content. *Lantana camara L.* has also been reported to cause mild stomach upset when unripe fruits or leaves are consumed, and its use is therefore limited and approached with caution.

For the majority of other WEPs, no adverse effects were reported. Nonetheless, community informants noted that excessive consumption of any single species, particularly unfamiliar or newly introduced WEPs, can occasionally result in mild stomach upset.

### Preference ranking of wild edible plants

Local communities in the Karamara forest patches exhibit clear preferences for specific WEPs based on taste, nutritional value, seasonal availability, and ease of collection. Participatory ranking exercises revealed species consistently identified as highly preferred, while others were less favored or reserved for periods of food scarcity.

Highly preferred species included leafy vegetables such as *Amaranthus caudatus* and *Moringa stenopetala*, valued for their palatability, versatility in cooked dishes, and year-round availability in home gardens or nearby fields. Similarly, fruits of *Ziziphus mauritiana, Tamarindus indica, Balanites aegyptiaca,* and *Ficus sycomorus* received high scores due to their sweet taste, cultural significance, and ability to provide quick nutrition during lean periods (Table 3).

**Table 3:** Preference ranking of WEPs in Karamara forest patches

Scientific name	Notes on preference	Rank
*Amaranthus caudatus* L	Highly palatable, cooked leafy vegetable	1
*Balanites aegyptiaca* (L.) Delile	Fruit, palatable and nutritionally useful	1
*Cordia africana* Lam	Fruit, seasonal, culturally valued	3
*Cucumis kelleri* (Cogn.) Ghebret. & Thulin	Raw fruit, seasonal availability	3
*Dovyalis abyssinica* (A.Rich.) Warb	Fruit, seasonal	3
*Ehretia cymosa* Thonn	Fruit, consumed during scarcity	3
*Euclea racemosa* L	Fruit, moderate preference	5
*Ficus sycomorus* L	Sweet fruit, highly preferred, eaten fresh	1
*Ficus vasta* Forssk	Fruit, moderately preferred	2
*Grewia mollis* Juss	Fruit, palatable and accessible	2
*Grewia schweinfurthii* Burret	Fruit, moderately preferred	4
*Grewia tenax* (Forssk.) Fiori	Fruit, good taste, seasonal availability	2
*Moringa stenopetala* (Baker f.) Cufod	Cooked leaves, highly valued	1
*Opuntia ficus-indica* (L.) Mill	Raw fruit, limited availability	5
*Osyris quadripartita* Salzm. ex Decne	Fruit, culturally valued	2
*Rosa abyssinica* R.Br. ex Lindl	Fruit, moderately used	3
*Rubus volkensii* Engl	Fruit, moderately preferred	2
*Senna occidentalis* (L.) Link	Fruit, used occasionally	5
*Tamarindus indica* L	Fruit, culturally and nutritionally valued	1
*Vachellia seyal* (Delile) P.J.H.Hurter	Gum/fruit, seasonal	3
*Ziziphus mauritiana* Lam	Sweet fruit, top preference	2
*Ziziphus mucronata* Willd	Fruit, culturally important	1

Preference scores are illustrative, based on taste, accessibility, nutritional value, and seasonal availability, with 1 = highest preference and 5 = lowest preference

Moderately preferred species included *Grewia mollis, Grewia tenax,* and *Carissa edulis*. These species were appreciated for their seasonal fruiting and contribution to household diets, although they were slightly less accessible or palatable compared to top-ranked species.

Less preferred species, such as *Opuntia ficus-indica, Acokanthera schimperi,* and *Cordia sinensis*, were generally used only during periods of food scarcity or when highly ranked species were unavailable. Species with potential toxicity, including *Datura stramonium* and *Lantana camara*, were deliberately avoided in routine consumption, reflecting local knowledge of adverse effects and cautious use. Leafy vegetables such as *Solanum nigrum* were moderately preferred, with proper cooking practices enhancing both safety and palatability.

### Direct matrix ranking of multi-purpose wild edible plants

WEPs in the Karamara forest patches serve a variety of functions beyond food, including medicinal use, fuelwood, fodder, construction, and cash income (Table 4). To assess the relative importance of species across these multiple uses, a Direct Matrix Ranking (DMR) exercise was conducted with local informants. Each species was scored against selected criteria, and cumulative scores reflected their overall multi-purpose utility.

**Table 4:** Direct matrix ranking of WEPs in Karamara forest patches

Scientific Name	FD	MD	FO	FW	CP	CN	IM	TS	R
*Acacia mellifera* (Vahl) Benth	3	3	5	4	5	4	2	26	1
*Acacia tortilis* (Forssk.) Hayne	3	2	4	4	5	4	2	24	2
*Acokanthera schimperi* (A.DC.) Schweinf	3	3	1	1	1	1	2	12	12
*Balanites aegyptiaca* (L.) Delile	4	3	2	3	4	3	3	22	4
*Canthium arboreum* S.Vidal	3	2	2	1	1	1	2	12	12
*Canthium coromandelicum* (Burm.f.) Alston	3	2	2	1	1	1	2	12	12
*Carissa edulis* (Forssk.) Vahl	4	3	2	1	1	1	3	15	9
*Cordia africana* Lam	4	3	3	3	3	4	3	23	3
*Cordia sinensis* Lam	3	2	2	2	4	2	2	17	7
*Datura stramonium* L	1	5	0	0	0	0	0	6	14
*Dovyalis abyssinica* (A.Rich.) Warb	3	2	1	1	3	1	2	13	11
*Ehretia cymosa* Thonn	3	3	2	1	4	1	2	13	11
*Ficus sycomorus* L	5	4	4	3	3	4	3	26	1
*Ficus vasta* Forssk	3	3	2	2	2	2	2	16	8
*Grewia mollis* Juss	4	3	2	1	3	1	2	16	8
*Grewia tenax* (Forssk.) Fiori	4	3	2	1	1	1	2	14	10
*Lantana camara* L	1	4	0	0	0	0	0	5	15
*Moringa stenopetala* (Baker f.) Cufod	5	5	2	1	1	1	3	18	6
*Opuntia ficus-indica* (L.) Mill.*	4	2	2	1	0	1	3	13	11
*Osyris quadripartita* Salzm. ex Decne	3	3	1	1	1	1	2	12	12
*Rhus vulgaris* Meikle	3	2	1	1	1	1	2	11	13
*Ricinus communis* L	2	3	1	2	3	1	2	14	10
*Rosa abyssinica* R.Br. ex Lindl	3	2	1	1	1	1	2	11	13
*Senegalia senegal* (L.) Britton	3	2	4	3	3	3	2	20	5
*Solanum nigrum* L	3	3	1	1	1	1	2	12	12
*Tamarindus indica* L	4	4	2	2	2	3	3	20	5
*Ximenia afra* Sond	3	2	1	1	1	1	2	11	13
*Ziziphus mauritiana* Lam	5	3	3	2	2	3	4	22	4
*Ziziphus mucronata* Willd	4	3	2	2	2	2	3	18	6
*Ziziphus spina-christi* (L.) Desf	5	4	3	2	2	3	3	23	3
TS	101	88	60	49	61	53	67		
R	1	2	5	7	4	6	3		

Direct matrix ranking with illustrative scores (0–5) across multiple use categories FD = Food, MD = Medicine, FO = Fodder, FW = Firewood, CP = Charcoal production, CN = Construction, IM = Income, TS = Total Score, R = Rank

The analysis identified several species as highly versatile and valuable across multiple dimensions. *Ficus sycomorus L.*, *Ziziphus mauritiana Lam.*, and *Ziziphus spina-christi (L.) Desf.* received the highest scores, owing to their edible fruits, medicinal properties, provision of fodder, and occasional use in construction or as shade trees. Similarly, *Moringa stenopetala (Baker f.) Cufod.* and *Acacia mellifera (Vahl) Benth.* were ranked highly for their combined nutritional, medicinal, and livestock fodder benefits.

Medium-ranking species, including *Grewia mollis Juss.*, *Carissa edulis (Forssk.) Vahl*, and *Cordia africana Lam.*, provided edible fruits as well as minor medicinal or construction uses, though their overall utility was lower compared to the top-ranked multipurpose plants.

Species with more limited multifunctional value, such as *Datura stramonium L.*, *Lantana camara L.*, and *Solanum nigrum L.*, scored low in the DMR, primarily due to toxicity concerns, restricted edible use, or limited applicability for fuel, fodder, or medicine.

### Most popularly utilized wild edible plants

The ethnobotanical survey of the Karamara forest patches documented a diverse assemblage of WEPs, with certain species emerging as most frequently utilized Based on consumption frequency, cultural importance, and household reliance during lean seasons.

Species of the genus *Amaranthus*, including *Amaranthus caudatus L.*, *A. dubius Mart. ex Thell.*, and *A. spinosus L.*, ranked highest in popularity due to their widespread use as cooked leafy vegetables and seeds (Table 5). These species serve as essential supplementary foods throughout the year, valued for their accessibility, nutritional content, and ability to complement grain-based diets.

**Table 5:** Most utilized WEPs from Karamara forest patches

Scientific name	Score
*Amaranthus caudatus* L	5
*Amaranthus dubius* Mart. ex Thell	5
*Amaranthus spinosus* L	5
*Ficus sycomorus* L	5
*Ziziphus mauritiana* Lam	5
*Ziziphus spina-christi* (L.) Desf	4.5
*Cordia africana* Lam	4.5
*Moringa stenopetala* (Baker f.) Cufod	4.5
*Ficus vasta* Forssk	4
*Carissa edulis* (Forssk.) Vahl	4
*Grewia mollis* Juss	4
*Vachellia seyal* (Delile) P.J.H.Hurter	4
*Tamarindus indica* L	3.5
*Grewia tenax* (Forssk.) Fiori	3.5
*Acacia mellifera* (Vahl) Benth	3.5
*Acacia tortilis* (Forssk.) Hayne	3.5
*Senegalia senegal* (L.) Britton	3.5
*Rhus vulgaris* Meikle	2.5
*Acokanthera schimperi* (A.DC.) Schweinf	2.5
*Opuntia ficus-indica* (L.) Mill	2.5
*Senna occidentalis* (L.) Link	2.5
*Commelina latifolia* Hochst. ex A.Rich	2.5
*Cucumis kelleri* (Cogn.) Ghebret. & Thulin	2.5
*Rubus volkensii* Engl	2
*Canthium arboreum* S.Vidal	2
*Dovyalis abyssinica* (A.Rich.) Warb	2
*Osyris quadripartita* Salzm. ex Decne	2
*Rosa abyssinica* R.Br. ex Lindl	2

Based on illustrative frequency of use and preference scores (0–5; 5 = highest use/popularity)

Fruits from trees such as *Ficus sycomorus L.*, *Ziziphus mauritiana Lam.*, *Ziziphus spina-christi (L.) Desf.*, and *Cordia africana Lam.* were commonly harvested and consumed raw. Their post-rain availability and integration into daily diets and cultural practices reinforce their role as key wild foods. Similarly, *Moringa stenopetala (Baker f.) Cufod.* was widely utilized for its nutrient-rich leaves, which are cooked or added to soups, reflecting both dietary significance and medicinal appreciation.

Other frequently used species included *Carissa edulis (Forssk.) Vahl*, *Grewia mollis Juss.*, *Ficus vasta Forssk.*, and *Vachellia seyal (Delile) P.J.H.Hurter*. These plants contribute not only edible parts but also additional household benefits such as fodder and minor income generation. Cultivated or semi-cultivated species, including *Opuntia ficus-indica (L.) Mill.* and *Amaranthus spp.*, were consistently cited for their reliability and ease of collection.

Conversely, species such as *Datura stramonium L.*, *Lantana camara L.*, and *Solanum nigrum L.* were less frequently used due to toxicity concerns or limited palatability, reflecting the local knowledge and risk-averse strategies employed in wild food selection.

### Newly identified wild edible plants in Karamara forest patches

The ethnobotanical survey of the Karamara forest patches documented a total of 42 wild edible plant (WEP) species, several of which were recorded for the first time in this locality, representing new additions to the regional ethnobotanical inventory. Newly identified species were defined as those not previously reported in district- or zone-level ethnobotanical studies of the Somali Region.

The newly documented WEPs include: *Vachellia seyal* (Delile) P.J.H.Hurter*, Grewia tenax* (Forssk.) Fiori*, Acacia mellifera* (Vahl) Benth.*, Tamarindus indica* L. *Amaranthus spinosus* L*.*, *Cucumis kelleri* (Cogn.) Ghebret. & Thulin, *Ehretia cymosa* Thonn*.*, *Senna occidentalis* (L.) Link,, and *Canthium coromandelicum* (Burm.f.) Alston. Informants reported these species as either additional foods (AF) or famine foods (Fam), with edible parts including fruits, leaves, seeds, or entire plants consumed either raw or cooked, depending on traditional preparation practices.

The discovery of these previously unrecorded species emphasizes the underexplored diversity of WEPs in the Karamara forest patches and highlights the importance of site-specific documentation. Notably, species such as *Amaranthus spinosus* and *Cucumis kelleri* are also cultivated in home gardens elsewhere, but their wild occurrence in Karamara represents a novel record.

### Implications of using wild edible plants in Karamara forest patches

The documented WEP species in Karamara play multiple roles in the local communities, from food security and nutrition to cultural, medicinal, and livelihood support.

Species such as *Amaranthus caudatus*, *A. dubius*, *A. spinosus*, *Opuntia ficus-indica*, and *Commelina latifolia* contribute essential micronutrients and dietary diversity to predominantly grain-based diets, particularly during lean seasons. Famine foods like *Rhus vulgaris*, *Carissa edulis*, *Cordia africana*, and *Cucumis kelleri* act as critical safety nets during droughts or periods of food scarcity (Table 6).

**Table 6:** Implications of the WEPs documented in the Karamara forest patches

Scientific name	Main implication
*Acacia tortilis* (Forssk.) Hayne	Food/ecosystem service
*Acokanthera schimperi* (A.DC.) Schweinf	Food
*Amaranthus caudatus* L	Food
*Balanites aegyptiaca* (L.) Delile	Food/income/ecosystem service
*Canthium arboreum* S.Vidal	Food/ecosystem service
*Carissa edulis* (Forssk.) Vahl	Food/famine
*Commelina latifolia* Hochst. ex A.Rich	Food
*Cordia africana* Lam	Food/ecosystem service
*Cucumis kelleri* (Cogn.) Ghebret. & Thulin	Food/famine
*Datura stramonium* L	Health/food (medicinal caution)
*Dovyalis abyssinica* (A.Rich.) Warb	Food/famine/income
*Ehretia cymosa* Thonn	Food
*Euclea racemosa* L	Food/health
*Ficus sycomorus* L	Food/ecosystem Service
*Grewia tenax* (Forssk.) Fiori	Food/income
*Lantana camara* L	Food/income (minor)
*Moringa stenopetala* (Baker f.) Cufod	Food/health/income
*Myrsine africana* L	Food/health
*Opuntia ficus-indica* (L.) Mill	Food/income
*Osyris quadripartita* Salzm. ex Decne	Food/health
*Rhus vulgaris* Meikle	Food
*Ricinus communis* L	Food/health
*Rosa abyssinica* R.Br. ex Lindl	Food/health
*Rubus volkensii* Engl	Food/famine
*Rumex nervosus* Vahl	Food/health
*Senna occidentalis* (L.) Link	Food/famine
*Solanum nigrum* L	Food/health
*Tamarindus indica* L	Food/income
*Vachellia seyal* (Delile) P.J.H.Hurter	Food/ecosystem service
*Ziziphus mucronata* Willd	Food/income

Several WEPs also possess bioactive compounds with antioxidant, antimicrobial, and anti-inflammatory properties. For example, *Moringa stenopetala*, *Ziziphus mauritiana*, and *Ricinus communis* are traditionally used to prevent nutrient deficiencies and treat minor ailments, highlighting their ethnomedicinal as well as nutritional significance.

Certain species, including *Balanites aegyptiaca*, *Ziziphus mucronata*, and *Moringa stenopetala*, have market potential, providing supplementary income that enhances household economic resilience. The multi-purpose nature of these plants serving as food, medicine, fuel, or construction material further strengthens their contribution to local livelihoods.

The Karamara forest patches also harbor species essential for ecosystem sustainability, such as *Cordia africana*, *Ficus sycomorus*, and *Acacia tortilis*, which maintain habitat structure and biodiversity while providing edible parts. Sustainable harvesting is critical to preserve WEP availability and associated ecological services. Threats such as deforestation, overgrazing, and invasive species jeopardize these resources, emphasizing the need for community-based management and conservation strategies.

### Priority ranking of factors affecting wild edible plants

The priority ranking exercise conducted with local informants identified key anthropogenic and environmental factors threatening the availability and sustainability of WEPs in the Karamara forest patches (Table 7).

**Table 7:** Priority ranking of factors affecting WEPs in the study area

Threating factors	Total	Rank
Agricultural extension	29	5th
Charcoal making	48	2nd
Construction and tools	23	7th
Fire wood collection	59	1st
Invasive alien species	25	6th
Overgrazing	39	3rd
Recurrent drought	35	4th

Firewood collection emerged as the most significant threat (ranked 1st; total score = 59), reflecting the heavy dependence of local households on forest resources for domestic energy. This reliance has led to unsustainable harvesting practices that compromise the regeneration and persistence of WEP species.

Charcoal production was ranked second (total score = 48) and is increasingly practiced as a cash-generating activity. The extraction of woody biomass for charcoal contributes substantially to forest degradation, particularly affecting multipurpose tree species that provide fruits, fodder, and medicinal resources.

Overgrazing ranked third (score = 39), with livestock pressure reducing natural regeneration and causing habitat disturbance. Similarly, recurrent drought (ranked 4th; score = 35) was recognized as a major environmental constraint, highlighting the vulnerability of WEP resources to climate variability in this dryland ecosystem.

Other threats, though ranked lower, include agricultural expansion (5th; score = 29), which leads to habitat conversion; invasive alien species (6th; score = 25), which compete with native WEPs; and use of plants for construction and tools (7th; score = 23), which exerts additional pressure on specific species.

### Conservation and management of wild edible plants in Karamara forest patches

The Karamara forest patches in Eastern Ethiopia harbor a rich diversity of WEPs that are vital to local food security, nutrition, livelihoods, and traditional medicine. However, these forest fragments face multiple anthropogenic and ecological pressures, including firewood collection, charcoal production, overgrazing, agricultural expansion, construction, invasive species (e.g., *Prosopis juliflora*), and recurrent drought. Such pressures threaten both the persistence of WEP species and the intergenerational transfer of ethnobotanical knowledge.

Effective conservation and management strategies are therefore essential to ensure the sustainable availability of these resources. The study revealed that WEP species vary in life forms, edibility patterns, and collection methods, which should inform targeted management plans. Herbs and leafy vegetables (e.g., *Amaranthus caudatus, Commelina latifolia*, *Solanum nigrum*) are predominantly harvested by plucking, while shrubs and trees (e.g., *Ficus sycomorus*, *Ziziphus mucronata*, *Cordia africana*) are mainly collected for fruits and gums via picking. Cultivated or semi-cultivated species such as *Moringa stenopetala* and *Opuntia ficus-indica* are often integrated into homegardens, offering opportunities for ex-situ conservation.

Community-based management is critical for sustainable use. Local households depend on WEPs as supplementary foods during lean seasons, sources of income through marketable species (e.g., *Opuntia ficus-indica*, *Tamarindus indica*, *Balanites aegyptiaca*), and materials for traditional medicine.

### IUCN red list status of wild edible plants

The conservation status of WEPs in Karamara Forest Patches is largely undocumented, complicating prioritization for sustainable management. A preliminary review indicates that most species have not been assessed by the International Union for Conservation of Nature (IUCN) Red List. Among those evaluated, species such as *Moringa stenopetala*, *Cordia africana*, *Tamarindus indica*, and *Balanites aegyptiaca* are classified as Least Concern (LC), highlighting the need for comprehensive assessment of unlisted species to inform conservation strategies (Table 8).

**Table 8:** Wild edible plant species from Karamara forest patches to their IUCN red list status

Scientific name	IUCN red list status
*Cordia africana* Lam	Least concern
*Opuntia ficus-indica* (L.) Mill	Least concern
*Acacia mellifera* (Vahl) Benth	Least concern
*Acacia tortilis* (Forssk.) Hayne	Least concern
*Senegalia senegal* (L.) Britton	Least concern
*Tamarindus indica* L	Least concern
*Vachellia seyal* (Delile) P.J.H.Hurter	Least concern
*Grewia tenax* (Forssk.) Fiori	Least concern
*Ficus sycomorus* L	Least concern
*Moringa stenopetala* (Baker f.) Cufod	Least concern
*Ximenia afra* Sond	Least concern
*Ziziphus mucronata* Willd	Least concern
*Ziziphus spina-christi* (L.) Desf	Least concern
*Ziziphus mauritiana* Lam	Least concern
*Balanites aegyptiaca* (L.) Delile	Least concern

### Wild edible plant knowledge differences among informant groups

Independent t-test analysis revealed variation in wild edible plant (WEP) knowledge among different informant groups in the Karamara forest patches (Table 9).

**Table 9:** Wild Edible Plant Knowledge among informant groups (independent t-test)

Parameters	Informant groups	*N*	*Mean* ± *SD*	*t-value*	*p–value*
Gender	Male	40	3.8 ± 1.9	4.4	*P* < *0.05*
	Female	24	2.1 ± 1.1		
Literacy level	Illiterate	45	4.2 ± 1.9	4.9	*P* < *0.05*
	Literate	19	2.0 ± 1.4		
Experience of informant	Key informant	24	5.7 ± 1.2	10.3	*P* < *0.05*
	General informant	40	2.2 ± 1.3		

Gender differences were evident, with male informants reporting higher mean number of WEPs (3.8 ± 1.9) than female informants (2.1 ± 1.1) (t = 4.4, p < 0.05). This pattern likely reflects men’s greater engagement in activities such as herding, hunting, and forest resource collection, which increases their exposure to diverse wild plant species.

Literacy status also influenced WEP knowledge. Illiterate participants demonstrated significantly greater knowledge (4.2 ± 1.9) than literate individuals (2.0 ± 1.4) (t = 4.9, p < 0.05), reflecting their stronger reliance on traditional ecological knowledge and closer interaction with local subsistence strategies. In contrast, literate individuals may depend more on market-based foods and less on wild resources.

Informant experience showed the most pronounced difference. Key informants, recognized locally for their expertise, reported substantially higher knowledge (5.7 ± 1.2) compared to general informants (2.2 ± 1.3) (t = 10.3, p < 0.05). This confirms the effectiveness of purposive selection of key informants in ethnobotanical studies, as they serve as custodians of traditional knowledge.

One-way ANOVA indicated a significant effect of age category on WEP knowledge (F = 50.54, df = 2, p < 0.05) (Table 10). Post-hoc comparisons revealed that elder informants possessed significantly greater knowledge than middle-aged (mean difference = 4.37, p < 0.001) and young informants (mean difference = 5.55, p < 0.001), while the difference between middle-aged and young participants was not statistically significant (mean difference = 1.18, p = 0.237). These findings highlight elders as the primary custodians of traditional knowledge, reflecting their lifelong experience, deeper engagement with natural resources, and cultural responsibility for knowledge transmission. The relatively lower knowledge among younger participants may result from declining dependence on wild resources, modernization, and increased reliance on market foods.

**Table 10:** Age Categories and Informant Knowledge of WEPs (One-Way ANOVA)

Source of variation	Df	SS	MS = SS/Df	F Ratio	*P-value*
Between groups	2	382.3	191.15	50.54	*P* < *0.05*
Residual (within)	61	230.7	3.78		
Total	63	613	194.93		

Df = degree of freedom, SS = Sum of Squares, MS = Mean of Square, Significant codes: 0.05

**Fig. 5:**
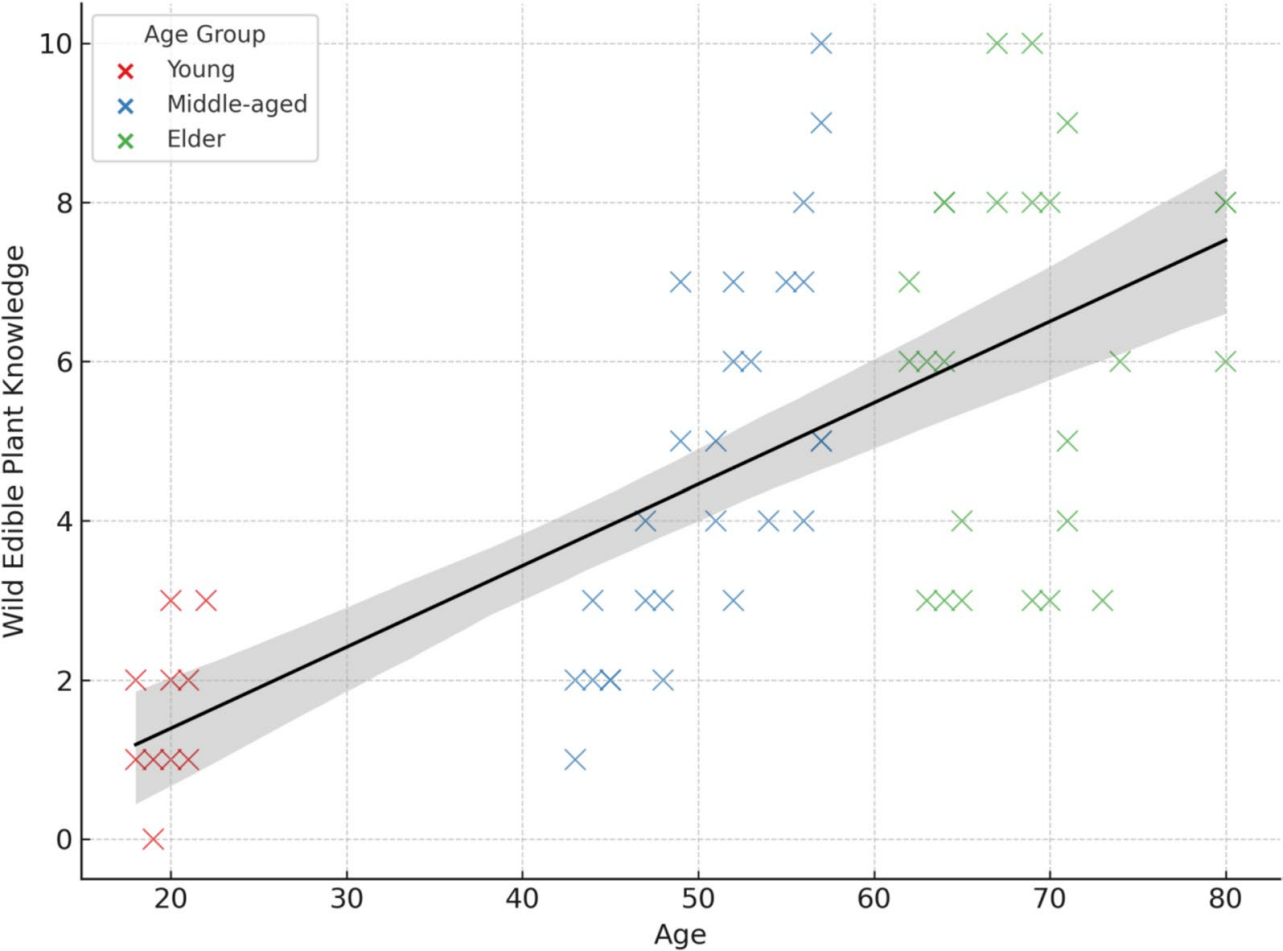
Correlation between age and wild edible plant knowledge of informants

Pearson correlation analysis further confirmed a strong positive relationship between informants’ age and WEP knowledge (R = 0.66, p < 0.001), indicating that older individuals consistently recognize and report more wild edible plant species (Fig. 5). Moreover, the application of the Botanical Ethnoknowledge Index (BEI) in the study area revealed significant variation in traditional medicinal plant knowledge across different informant groups, influenced by sociocultural factors such as gender, informant status, education, and age.

### Indigenous knowledge transfer and practices of wild edible plants

**Fig. 6:**
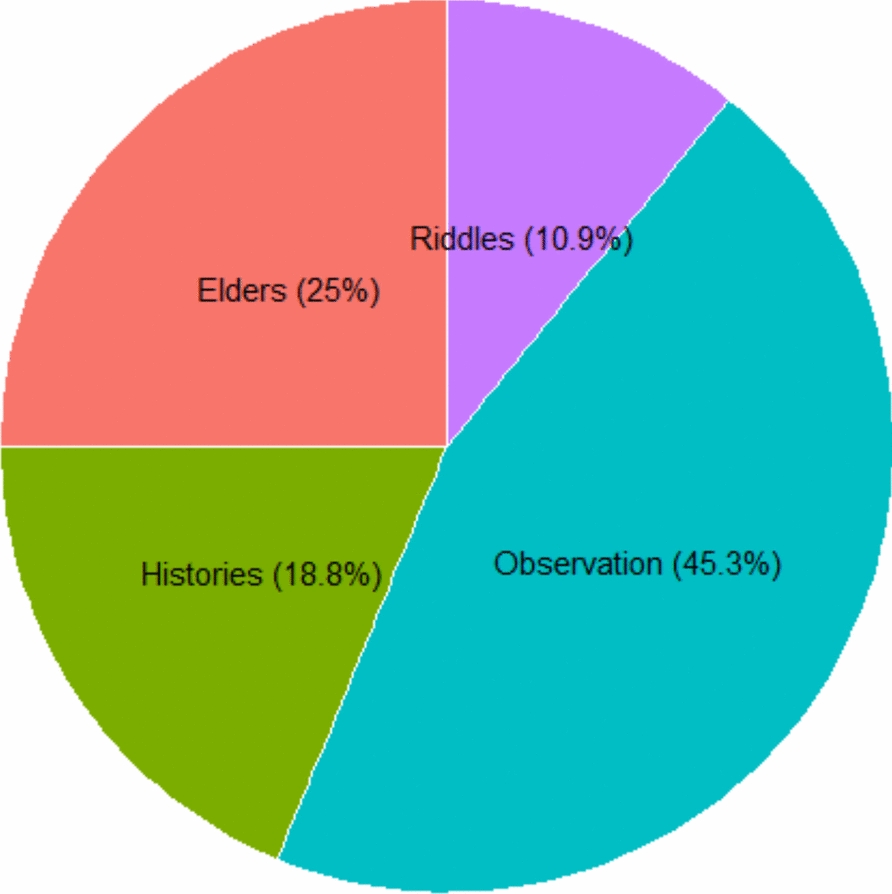
Indigenous knowledge transfer of wild edible plants

The use of WEPs in the Karamara forest patches is deeply rooted in traditional knowledge systems, which govern species identification, harvesting, preparation, and consumption. Among the informants, 29 reported acquiring knowledge through direct observation, 16 learned discreetly from elders at an advanced age, 12 gained knowledge via oral histories, and 7 learned through local stories or riddles shared during evening gatherings (Fig. 6).

Indigenous knowledge is primarily transmitted orally across generations, often through family elders, community leaders, and experienced gatherers. This transmission encompasses recognizing edible species, determining which plant parts are safe for consumption, understanding seasonal availability, and identifying potentially toxic species. For example, *Moringa stenopetala*, *Commelina latifolia*, *Solanum nigrum*, and *Amaranthus caudatus* require specific processing methods that are learned through observation and hands-on demonstration.

Traditional practices also include sustainable harvesting techniques designed to minimize plant damage and ensure future availability. Methods such as selective plucking, picking, and digging are guided by community rules on quantity and timing, particularly for famine foods like *Solanum nigrum*, *Rubus volkensii*, and *Senna occidentalis*. These practices reduce the risk of overexploitation and support the long-term sustainability of WEP resources.

Preparation methods reflect sophisticated indigenous knowledge aimed at improving palatability and safety. Leaves, fruits, and resins are processed using specific techniques to remove bitterness, reduce toxicity, or enhance flavor. For instance, tubers of *Ricinus communis* and *Solanum nigrum* are typically boiled or roasted before consumption, while resins from Acacia tortilis and Vachellia seyal are used sparingly for flavoring or medicinal purposes.

## Discussion

### Diversity of wild edible plants in the karamara forest patches

The documentation of 42 WEPs species belonging to 32 genera and 24 families in the Karamara forest patches reflects a substantial contribution of forest biodiversity to local food systems. The prominence of Fabaceae, represented by six species, followed by Moraceae, Boraginaceae, and Malvaceae, aligns with other ethnobotanical studies in Ethiopia and elsewhere, where these families frequently serve as important sources of edible fruits, leaves, and versatile resources [29–41]. Fabaceae’s dominance is likely due to its ecological adaptability, widespread occurrence in semi-arid regions, diverse food applications, and nitrogen-fixing ability. For instance, studies in Simada District [57], Dibatie District [40], and Midakegn District [13] also highlighted Fabaceae and Moraceae among the dominant families, underscoring their ecological adaptability and cultural salience [42–54]. However, the total number of species recorded in Karamara (42) is lower than the 50–64 species reported from areas such as Soro District [55] and Dibatie District [40]. This discrepancy may be attributed to ecological differences, forest fragmentation, or variation in ethnobotanical knowledge systems [56–60].

Globally, similar richness patterns are evident. In Kohistan, Pakistan, 64 WEP species were documented, with Rosaceae, Lamiaceae, and Fabaceae being the dominant families [58]. Likewise, in Lalku Valley, Swat, Pakistan, 60 species from 31 families were reported, again with Rosaceae at the forefront [59]. In Africa, the miombo woodlands of Tanzania yielded 63 edible species, further confirming the role of diverse woodland ecosystems in sustaining local diets [60]. Compared with these global contexts, the Karamara inventory represents moderate species richness, suggesting either ecological constraints or changes in foraging practices that limit reliance on certain taxa.

The presence of semi-cultivated species such as *Amaranthus spp.*, *Moringa stenopetala*, and *Opuntia ficus-indica* demonstrates a local trend toward integrating wild resources into managed systems. Similar dynamics were reported in Midakegn, Ethiopia, where communities practice semi-domestication as a food security strategy [13, 29]. This reflects an adaptive response to resource scarcity and seasonal variability, where households actively manage useful WEPs to secure a reliable supply. The strong cultural significance of species such as *Ficus sycomorus*, *Moringa stenopetala*, and *Carissa edulis* highlights the role of ethnobotanical knowledge in shaping species preference, protection, and intergenerational transfer of use practices [57].

The dominance of certain families indicates both ecological suitability to semi-arid conditions and cultural valuation. The representation of unique taxa from single-species families, including *Ximenia afra* and *Balanites aegyptiaca*, underlines the specialized contributions of less abundant species to food diversity. The slightly lower richness of Karamara relative to other Ethiopian regions suggests potential pressures such as deforestation, agricultural expansion, and erosion of traditional knowledge. These findings highlight the vulnerability of forest patches, which are increasingly threatened by anthropogenic pressures.

### Life forms of wild edible plant species

The analysis of life forms of WEPs in the Karamara forest patches revealed that shrubs were the most dominant growth form, followed by trees and herbs. This result highlights their ecological adaptability and accessibility for local communities. Species such as *Carissa edulis*, *Grewia spp.*, and *Ziziphus spp.* are widely known across semi-arid regions of Ethiopia for their drought tolerance, ease of harvesting, and multifunctional uses [50, 57]. This predominance of shrubs aligns with findings from Simada, Mieso and Lowland Ethiopia Districts, where shrubs also represented the largest proportion of wild edibles [30, 50, 57], and Dibatie District, where shrubs accounted for a significant share of WEP diversity [40]. Similarly, in the Hadiya Zone, shrubs were highly valued for their edible fruits and adaptability to disturbed landscapes [55]. The recurrence of shrubs as the most common life form across multiple Ethiopian landscapes indicates both ecological resilience and cultural preference for shrub by fruit-bearing species.

Trees, the seond dominant of WEPs in Karamara, with species such as *Cordia africana*, *Ficus sycomorus*, *Tamarindus indica*, *Moringa stenopetala*, and *Balanites aegyptiaca* playing key nutritional, cultural, and ecological roles. Tree species provide perennial resources, shade, and are often deeply embedded in traditional practices. This finding is in line with studies conducted in the Midakegn, Chilga, and Delanta districts, which revealed that trees play a major role in local food systems by providing fruits, seeds, and leaves [13, 38, 39]. Globally, trees are also prominent in WEP inventories for instance, in Tanzania’s miombo woodlands, tree species such as *Uapaca kirkiana* and *Strychnos spp.* are highly significant [60], while in northern Pakistan, fruit-bearing trees including *Prunus* and *Juglans* species contribute substantially to local diets [59]. This finding suggests that trees serve as long-term and reliable sources of food and other resources across diverse ecological and cultural settings.

Herbs, though the least represented life form in Karamara, remain nutritionally vital. Species such as *Amaranthus spp.*, *Rumex nervosus*, and *Solanum nigrum* are important leafy vegetables rich in vitamins and minerals, particularly consumed during the rainy season when fresh greens are plentiful. Despite their relatively lower abundance, herbs contribute significantly to dietary diversity and seasonal food security. This pattern is consistent with reports from the Hadiya Zone, where herbs played a complementary role in household diets during specific seasons [55]. Similarly, in India and China, wild leafy greens such as *Chenopodium album* and *Portulaca oleracea* are crucial sources of micronutrients and are traditionally consumed during monsoons or spring seasons [61–63]. In Europe and North America, wild herbs such as nettles (*Urtica dioica*) and dandelions (*Taraxacum officinale*) are also valued for their nutritional and medicinal properties [64–67], suggesting a global recognition of the importance of herbs as supplementary but nutrient-dense food resources.

The dominance of shrubs and trees reflects the adaptation of local communities to semi-arid environments where perennial and woody species offer more reliable food sources than short-lived herbs. However, the seasonal and nutritional value of herbs highlights their irreplaceable role in dietary resilience. The life form distribution in Karamara mirrors broader ethnobotanical patterns across Ethiopia, Africa, and Asia, where shrubs and trees provide staple wild fruits and seeds, while herbs serve as seasonal supplements.

### Edible parts and modes of consumption

The analysis of edible parts and modes of consumption of WEPs in the Karamara forest patches demonstrates that fruits are the most widely consumed component, followed by leaves, seeds, gums, whole plants, and flower nectar. Fruits, used from 69% of the species, dominate local diets due to their availability, palatability, and convenience of consumption. Species such as *Carissa edulis*, *Cordia africana*, *Grewia spp.*, and *Ziziphus spp.* are highly valued for their sweet and energy-rich fruits [30, 50, 57]. This dominance of fruits is consistent with findings from other parts of Ethiopia, such as the Simada District, where fruits constituted the primary edible parts in 62% of the documented WEPs [57], and Yalo, Kebridehar and Dibatie Districts, where fruits were also reported as the most frequently consumed part [32, 40, 56]. Globally, fruit predominance is a recurring pattern: in Pakistan’s Kohistan region, 61% of WEPs were fruit-bearing species [59], while in northern Tanzania, fruit species such as *Strychnos* and *Uapaca* were central to local diets [60]. These similarities indicate the widespread reliance on wild fruits as readily available, portable, and nutrient-rich food sources, particularly in rural and dryland ecosystems.

Leaves were the second most important edible part in Karamara, contributing 14% of species, notably *Amaranthus spp.*, *Moringa stenopetala*, and *Solanum nigrum*. Leafy WEPs are often cooked as vegetables or incorporated into sauces and soups, supplying essential vitamins, minerals, and proteins. Their seasonal availability during rainy periods enhances dietary diversity and micronutrient intake. Similar reliance on leafy greens has been reported in the Hadiya Zone and Konso, Ethiopia [48, 55], and globally in Asia. For example, in eastern India, wild leafy greens such as *Chenopodium album* and *Portulaca oleracea* form a crucial part of rural diets [61], while in China, traditional consumption of wild leafy vegetables remains central to nutritional security and culinary heritage [62, 63]. In Europe, species like *Urtica dioica* and *Taraxacum officinale* are similarly valued for their nutrient richness [64, 65, 68]. This convergence across continents demonstrates the broad nutritional importance of wild leafy vegetables as supplementary but indispensable components of local diets.

Seeds, reported in *Amaranthus spp.*, accounted for 7% of WEPs in Karamara. Their preparation as porridge or cereal mixes reflects the blurred boundary between wild and domesticated food systems, as Amaranthus is both semi-cultivated and wild-harvested. Similar patterns of integrating semi-domesticated seeds into local diets have been noted in Meinit and Midakegn, Ethiopia [13, 51], and in South Asia, where wild grains such as *Echinochloa colona* are consumed during food shortages [61]. Such practices emphasize the role of WEP seeds as famine foods and as bridges between subsistence and cultivated agriculture.

Gums, obtained from *Acacia tortilis*, *Senegalia senegal*, and *Vachellia seyal*, were reported as snacks and energy sources. Their cultural importance, particularly among children and herders, highlights their role as traditional dryland foods. Gum consumption has also been documented in other semi-arid parts of Ethiopia [30, 50] and across the Sahel, where Acacia gums are integral to both diets and trade [69, 70]. The persistence of gum use demonstrates adaptation to harsh environments where few other food options are available during long fieldwork or mobility.

Whole plant consumption, recorded for *Rumex nervosus* and *Rubus volkensii*, represents a coping strategy during acute food scarcity. Similarly, famine foods involving whole herbs or roots are widely reported in sub-Saharan Africa and Asia [59, 62, 71]. Though minor in proportion, their role in survival contexts is disproportionately important. Flower nectar, noted for *Datura stramonium*, was the least reported category, reflecting its limited nutritional role but cultural familiarity, a pattern also observed in ethnobotanical studies from South Asia [58, 72].

Modes of consumption revealed that the majority of WEPs (81%) were consumed raw, primarily fruits and gums, while only 19% were cooked, reflecting a preference for minimally processed wild foods. This trend aligns with reports from Ethiopia’s Soro District [55], Pakistan [58], and India [61], where fruits dominate raw consumption and leafy greens require cooking. The predominance of raw consumption highlights both convenience and the immediacy of energy intake in subsistence contexts.

These findings emphasize that the choice of edible parts and their methods of preparation are shaped by both ecological availability and cultural practices. Fruits and leaves provide the bulk of daily dietary contributions, while seeds, gums, and whole plants act as resilience foods during scarcity. The reliance on raw consumption also indicates the limited need for processing, fitting the mobility and subsistence patterns of agro-pastoralist communities.

### Collection methods of wild edible plants

The results from the Karamara forest patches indicate that local communities employ simple yet effective collection methods primarily picking and plucking closely aligned with the edible parts and life forms of plants. Picking, practiced for 71.4% of species, was the most common method, particularly for fruit-bearing trees and shrubs such as *Cordia africana*, *Ficus spp.*, *Grewia spp.*, and *Ziziphus spp.*. This technique, involving hand collection, branch shaking, or gathering fallen fruits, is minimally destructive and sustainable across multiple fruiting seasons. Similar findings have been reported in other parts of Ethiopia, where fruits are primarily collected by hand due to their accessibility and cultural preference for fresh consumption [29, 31, 43, 47]. Comparable practices are documented in other African contexts, such as Tanzania, where communities harvest fruits of *Uapaca kirkiana* and *Strychnos spp.* directly from trees, often involving children in collection [60]. Globally, hand-picking is also the dominant strategy in Asia and South America; in India, wild fruits like *Ziziphus mauritiana* are collected through climbing and branch shaking [61], while in Mexico and Brazil, fruits of cacti and palms are commonly harvested by manual picking [73, 74]. This consistency across regions demonstrates that fruit harvesting through simple manual techniques is both a culturally embedded and ecologically sustainable practice.

Plucking, reported for 28.6% of species in Karamara, was mainly associated with leafy vegetables (*Amaranthus spp.*, *Solanum nigrum*, *Commelina latifolia*) and gums from leguminous trees (*Acacia tortilis*, *Senegalia senegal*, *Vachellia seyal*). While effective for obtaining tender shoots and exudates, plucking can be more labor-intensive and potentially destructive if not managed carefully, as repeated removal of young leaves may weaken plants. Similar concerns have been noted in the East shewa Zone of Ethiopia, where frequent harvesting of wild leafy vegetables risked overexploitation of localized populations [42]. In Pakistan’s Kohistan region, plucking of edible leaves and shoots is also widespread, though sustainable practices are increasingly threatened by intensified harvesting pressures [58]. In China, excessive harvesting of wild leafy greens such as *Portulaca oleracea* has raised ecological concerns, prompting calls for domestication and cultivation [63, 75]. These similarities suggest that, while plucking is nutritionally valuable for obtaining leafy vegetables and gums, maintaining ecological sustainability require appropriate management strategies to prevent resource degradation.

The predominance of picking over plucking reflects both the ecological availability of fruits in semi-arid forest patches and cultural preferences for fruits as primary WEPs. Picking, as a low-impact method, is inherently more sustainable than plucking, which carries risks of plant damage if conducted indiscriminately. The gendered and generational dimensions of collection where fruits are often picked by children and women, while gum collection is common among herders also highlight the social embeddedness of harvesting practices, a trend similarly observed in Ethiopia [30, 41, 44, 57] and across Africa and Asia [59, 60, 71].

### Consumption period of wild edible plants

The classification of WEPs in the Karamara forest patches into additional foods (64.3%) and famine foods (35.7%) reflects the dual role of these resources in sustaining local diets. The predominance of additional foods, including *Amaranthus spp.*, *Moringa stenopetala*, *Opuntia ficus-indica*, and *Balanites aegyptiaca*, highlights the significance of WEPs as routine dietary supplements that enhance nutritional diversity and food security. These species, especially leafy vegetables and fruits, are typically consumed during the rainy and post-rainy seasons when availability is high, echoing findings from other Ethiopian studies. For example, in Yeki and Dibatie District, most WEPs were reported as supplementary foods consumed seasonally to complement staples [2, 40], while in Simada District, fruits and leafy greens also dominated the category of additional foods [57]. Such patterns emphasize that WEPs are not marginal but integral to household nutrition, particularly in semi-arid regions where agricultural outputs are variable.

The considerable proportion of famine foods (35.7%), such as *Cordia africana*, *Rumex nervosus*, *Datura stramonium*, and *Solanum nigrum*, underlines their role as critical coping mechanisms during droughts, crop failures, or other periods of acute food insecurity. This aligns with observations in other Ethiopian regions, where famine foods often bridge seasonal food gaps [13, 21, 34, 36, 39]. In Africa more broadly, reliance on famine foods is well-documented; in Tanzania, species such as *Dioscorea* tubers and wild fruits serve as survival foods during droughts [60]. Similarly, in arid parts of India, famine foods like wild cucurbits and leafy greens are consumed in periods of scarcity [61], while in Pakistan’s Kohistan, *Berberis* and *Ziziphus* fruits function as emergency food resources [58, 72, 76]. Across Asia and Africa, therefore, famine foods consistently provide safety nets during times of crisis.

The high proportion of famine foods in Karamara reflects both ecological realities of the Somali Region and cultural resilience strategies. Communities actively retain knowledge of which plants are edible during scarcity, transmitting this knowledge across generations. However, the heavy reliance on famine foods in times of stress also signals vulnerability, as overdependence may lead to nutritional deficiencies or overharvesting of certain species. Globally, similar challenges have been reported: in rural China, some famine foods are associated with toxicity risks when improperly prepared [62, 63, 77], while in Europe, historical use of famine foods such as acorns and wild roots declined due to their inferior taste and energy content compared to cultivated crops [67, 78].

### Seasonal availability of wild edible plants

The seasonal availability of WEPs in the Karamara forest patches illustrates how communities strategically depend on plant diversity to manage fluctuations in food resources. Herbaceous and leafy species such as *Amaranthus caudatus*, *Commelina latifolia*, and *Solanum nigrum* dominate during the rainy season, when fresh greens are abundant and incorporated into daily diets. This pattern is consistent with observations from the Awi, Bullen, Midakegn, and Simada districts in Ethiopia, where seasonal leafy vegetables contributed essential micronutrients and enhanced dietary diversity during the rainy season [13, 35, 36, 57]. Similarly, in Pakistan’s Kohistan and India’s arid zones, wild leafy greens such as *Chenopodium* and *Portulaca* are consumed primarily in the rainy season, linking their availability with agricultural lean periods [58, 61]. Across Africa and Asia, seasonal leafy WEPs thus play a complementary role in food security by aligning their peak availability with times when cultivated vegetables are scarce.

Fruiting species including *Cordia africana*, *Ziziphus* spp., *Tamarindus indica*, and *Balanites aegyptiaca* reach their peak after the main rainy season, providing energy-rich foods at a critical time when cereal stocks from the harvest are declining. Similar trends were reported in Mieso, Lowland Ethiopia and Dire Dawa districts of Ethiopia, where fruits of *Grewia*, *Ziziphus*, and *Cordia* were heavily consumed post-rains as transitional foods bridging the gap to the next harvest [30, 41, 50]. In Tanzania’s miombo woodlands, post-rainy season fruiting species such as *Uapaca kirkiana* and *Strychnos spp.* fulfill comparable roles [60]. In South America, seasonal fruits like those of palms and cacti also provide dietary staples during transitional seasons [79]. The timing of fruit availability thus highlights the ecological alignment between the fruiting cycles of WEPs and the food requirements of households.

Drought-tolerant species such as *Acacia mellifera*, *Acacia tortilis*, *Senegalia senegal*, and *Vachellia seyal* serve as critical resources during prolonged dry spells. These species, providing gums, seeds, and leaves, function as emergency foods and grazing reserves, demonstrating their ecological and cultural importance in arid and semi-arid systems. Comparable observations are noted in northern Ethiopia and the Somali Region, where *Acacia* and *Balanites* species are relied upon as famine reserves [32]. In arid South Asia, drought-tolerant taxa such as *Ziziphus mauritiana* and *Prosopis cineraria* play analogous roles in buffering food shortages [61, 80]. The persistence of these hardy species underlines their role as keystone famine foods, essential to resilience strategies in resource-scarce landscapes.

Opportunistic and famine species such as *Rumex nervosus*, *Rubus volkensii*, *Dovyalis abyssinica*, and *Datura stramonium* are harvested sporadically during acute scarcity. These species represent emergency coping strategies, reflecting deep ethnobotanical knowledge of survival foods. This pattern is consistent with reports from Ethiopia’s Metema, Bullen and Yalo, where wild tubers and weedy greens are consumed during droughts [36, 52, 56]. Globally, similar opportunistic uses of wild plants are documented: in rural China, famine foods such as *Polygonum* species are consumed during scarcity [62, 81], while in Europe, historical accounts show reliance on acorns and wild roots during famines [78]. These cross-cultural similarities stress the universal role of WEPs as crucial fallback resources in human subsistence strategies.

### Marketability of wild edible plants

The finding that 28% of WEPs documented in Karamara forest patches have market value highlights their dual role as both subsistence foods and sources of income. Marketable species were dominated by fruit-bearing plants such as *Ficus sycomorus*, *Ziziphus spp.*, *Grewia spp.*, and *Tamarindus indica*, reflecting consumer demand for palatable, nutrient-rich, and easily transportable products. Fruits like *Ziziphus mauritiana* and *Grewia tenax* are particularly valued for their sweetness and shelf life, making them highly traded in local markets. This trend is consistent with studies in Ethiopia’s Metema, Goba and Dibatie Districts, where fruits accounted for the majority of marketed WEPs [40, 45, 52], and in Kebridehar and Simada Districts, where species such as *Cordia africana* and *Balanites aegyptiaca* were reported as significant trade commodities [32, 57]. These equivalents emphasize the key role of fruits in supporting both household nutrition and income generation.

Leafy vegetables, particularly *Moringa stenopetala* and *Amaranthus spp.*, also feature prominently in markets during the rainy season when fresh supply is abundant. Their market presence demonstrates how seasonal availability intersects with economic opportunities, a pattern similarly observed in the Hadiya Zone of Ethiopia, where wild leafy vegetables were sold fresh in periodic markets [55]. Comparable findings are reported globally: in Pakistan, *Amaranthus* and *Ziziphus* fruits are among the most frequently traded WEPs [24, 58, 59], while in India, leafy greens such as *Chenopodium album* are sold in village markets as seasonal delicacies [61, 82]. In rural China, wild vegetables like *Portulaca oleracea* and fruits such as *Schisandra chinensis* are commercialized in both local and urban markets, linking biodiversity to rural livelihoods [62, 63, 83]. These similarities reflect the widespread economic potential of WEPs when integrated into local food systems.

The marketability of WEPs is shaped by ecological and social factors, including species abundance, cultural acceptability, and proximity to trade networks. In Karamara, species harvested from accessible forest edges and home gardens are more likely to be commercialized than those from remote patches, reflecting the importance of landscape accessibility. Comparable dynamics are reported in Africa’s miombo woodlands, where proximity to roads determines the likelihood of wild fruits entering markets [60]. In Europe and North America, historical records show that only species with clear cultural recognition and safety, such as berries and nuts, entered local markets [67]. This illustrates how cultural valuation and trust shape the commercialization potential of WEPs alongside ecological abundance.

### Adverse effects associated with consumption of wild edible plants

The findings from the Karamara forest patches show that while most WEPs are consumed safely and contribute positively to food security and nutrition, some species pose potential health risks if improperly prepared or consumed in excess. For example, *Datura stramonium* was identified as toxic due to its tropane alkaloid content, consistent with global reports of poisoning and hallucinogenic effects associated with this species [84]. Its restricted use in ritual and medicinal contexts in Karamara reflects similar patterns observed in India and Pakistan, where the plant holds cultural significance but is deliberately avoided as a staple food [85].

Similarly, *Solanum nigrum* represents a plant with dual status: safe and nutritious when cooked, but potentially harmful if consumed raw due to solanine content. This aligns with Ethiopian studies from Soro, Dibatie, Metema and Quara districts, which also reported the need for proper preparation to mitigate toxicity [21, 40, 55]. In Asia, particularly China, *S. nigrum* is widely used as a leafy vegetable, but only after boiling, which reduces toxic glycoalkaloids [63]. Comparable findings are reported in Europe, where traditional culinary knowledge emphasizes processing methods for nightshade family plants [67]. These cross-regional similarities highlight the importance of indigenous knowledge in promoting food safety and sustaining dietary diversity.

Another species noted for potential risks was *Lantana camara*, which may cause mild gastrointestinal upset when unripe fruits or leaves are ingested. While its consumption as food is rare, its accidental ingestion has been linked to livestock poisoning in East Africa [86, 87]. Similar cautionary reports exist from Latin America, where unripe *Lantana* fruits are occasionally mistaken for edible berries [88, 89]. This underlines the importance of distinguishing between culturally validated food species and those with ambiguous or dangerous status.

The broader implication of these findings is the necessity of safeguarding local knowledge systems that guide safe use of WEPs. Communities in Karamara, like those in other regions, rely on accumulated ethnobotanical knowledge to distinguish safe from unsafe species, determine preparation methods, and avoid harmful doses. The decline of such knowledge due to modernization and reduced intergenerational transfer raises concern, as loss of traditional practices could increase the risk of accidental poisoning [2, 45, 56]. Promoting food literacy, documenting preparation practices, and integrating toxicological screening into ethnobotanical research are therefore essential for ensuring both safety and sustainability.

From a policy perspective, incorporating WEPs into nutrition and food security strategies requires not only recognition of their dietary and economic value but also careful consideration of their potential adverse effects. This dual approach promoting safe, nutrient-rich WEPs while raising awareness of toxic species could enhance their role in combating malnutrition and food insecurity across Ethiopia and comparable dryland ecosystems worldwide.

### Preference ranking of wild edible plants

The preference ranking of WEPs in the Karamara forest patches reflects a strong interplay between cultural valuation, nutritional importance, and ecological accessibility. Highly ranked species such as *Amaranthus caudatus*, *Moringa stenopetala*, *Ziziphus mauritiana*, *Tamarindus indica*, and *Ficus sycomorus* were favored for their taste, nutritional value, versatility, and availability. This aligns with ethnobotanical studies from other Ethiopian regions, such as Konso, Yeki, Soro and Simada districts, where leafy vegetables and sweet fruits were consistently prioritized for daily diets and seasonal supplementation [2, 48, 55, 57]. Globally, similar patterns are evident: in Pakistan, fruits of *Ziziphus* and *Grewia* species are preferred for their sweetness and energy content [58], while in India and China, leafy greens like *Amaranthus* and *Solanum* are highly valued for their micronutrient contributions [61, 63]. These findings highlight the universal role of palatability and nutritional density in shaping WEP preferences.

Moderately preferred species such as *Grewia mollis*, *Grewia tenax*, and *Carissa edulis* were valued for their seasonal fruiting and contribution to household nutrition, though limited by accessibility or less favorable taste compared to top-ranked species. Comparable observations have been reported in Africa’s Sahel region, where *Grewia* fruits serve as supplementary foods but are less prioritized compared to *Ziziphus* or *Tamarindus* [90, 91]. In Latin America, similar seasonal reliance on moderately preferred fruits like *Byrsonima crassifolia* reflects the role of ecological seasonality in shaping preferences [92, 93].

Less preferred species, such as *Opuntia ficus-indica* and *Cordia sinensis*, were mostly consumed during food scarcity, indicating their role as fallback or famine foods. This aligns with observations from other Ethiopian drylands, where Opuntia and Rumex nervosus are considered emergency foods rather than preferred components of the regular diet [30, 32, 41, 50, 56].

Similar fallback roles are reported in rural India with wild yams (*Dioscorea spp.*) and in Tanzania’s miombo woodlands, where less palatable fruits are consumed only during lean periods [60]. The avoidance of species such as *Datura stramonium* and *Lantana camara* due to toxicity further illustrates the depth of local ecological knowledge in differentiating safe from harmful plants, echoing reports from Europe and North America where traditional food cultures deliberately excluded toxic taxa despite their availability [65–67].

### Direct matrix ranking of multi-purpose wild edible plants

The results of the Direct Matrix Ranking (DMR) in Karamara forest patches highlight the multifunctionality of WEPs and their integral role in local livelihoods. Species such as *Ficus sycomorus*, *Ziziphus mauritiana*, and *Ziziphus spina-christi* were identified as highly versatile, providing edible fruits, medicinal benefits, fodder, shade, and occasional construction material. Their high cumulative scores reflect both ecological abundance and cultural valuation, consistent with findings from Ethiopia’s Yeki, Raya, and Burji districts, where multifunctional WEPs were similarly prioritized for nutritional, medicinal, and economic uses [2, 31, 37].

*Moringa stenopetala* and *Acacia mellifera* were also ranked highly for their combined roles in food, medicine, and livestock fodder, illustrating the critical contribution of nutrient-rich leafy vegetables and drought-tolerant legumes to both human and animal nutrition. Comparable multifunctional species are documented in Pakistan and India, where *Moringa oleifera* and *Acacia nilotica* are valued for edible leaves, traditional medicine, and fodder provision [58, 61]. In Africa’s semi-arid regions, *Acacia* species serve similar multipurpose roles, underpinning household resilience to environmental stress and food insecurity [94, 95]. These similarities highlight the worldwide importance of multifunctional WEPs in supporting rural livelihoods, especially in dryland ecosystems.

Medium-ranking species, such as *Grewia mollis*, *Carissa edulis*, and *Cordia africana*, contribute primarily through edible fruits and minor medicinal or construction uses. While their overall utility is lower than top-ranked species, they nonetheless complement household diets and provide secondary income, consistent with observations from Ethiopian and Tanzanian ethnobotanical studies [21, 33, 55, 60]. In other regions of Asia and Latin America, analogous species serve as supplementary resources during seasonal shortages or as minor cash crops, highlighting their indirect role in food security [63, 92].

Species with restricted multifunctionality, including *Datura stramonium*, *Lantana camara*, and *Solanum nigrum*, scored low in the DMR due to toxicity, limited edible use, or minimal application in fuel, fodder, or construction. This demonstrates the selective use of WEPs informed by indigenous knowledge of safety and utility, reflecting a risk-aware approach to resource management. Similar patterns are observed in Europe and North America, where culturally recognized species are prioritized for multiple uses, while toxic or low-utility species are avoided or restricted to medicinal applications [66, 67, 84, 92].

### Most popularly utilized wild edible plants

The ethnobotanical survey of Karamara forest patches revealed that certain WEPs are more frequently utilized, reflecting a combination of accessibility, nutritional value, cultural significance, and seasonal reliability. Species of the genus *Amaranthus*, including *A. caudatus*, *A. dubius*, and *A. spinosus*, emerged as the most popular leafy vegetables, consumed throughout the year either as cooked greens or seeds. Their extensive use is consistent with findings from other Ethiopian regions, including the Meinit and Yeki districts, where *Amaranthus* species play a key role in household nutrition and dietary supplementation [2, 51]. Internationally, *Amaranthus* species are similarly highly valued in Pakistan and India, where they provide essential vitamins and minerals, complementing cereal-based diets and contributing to food security during lean periods [72, 96].

Fruit-bearing trees, including *Ficus sycomorus*, *Ziziphus mauritiana*, *Ziziphus spina-christi*, and *Cordia africana*, were also prominently utilized. These species are integrated into daily diets and cultural practices, particularly post-rain, reinforcing their role as both nutritional and cultural staples. Comparable patterns are reported in other African contexts, such as the Sahel and miombo woodlands, where *Ziziphus* and *Ficus* fruits serve as both household foods and minor income sources [60, 90]. In Asia, fruits of *Ziziphus* and *Ficus* species similarly provide emergency nutrition and are widely marketed in local rural markets, highlighting their cross-cultural and ecological significance [62, 85, 97].

*Moringa stenopetala* was commonly reported for its nutrient-dense leaves, used in cooking or added to soups, highlighting its combined nutritional and medicinal value. This aligns with the widespread use of *Moringa* species in Ethiopia, India, and Pakistan, where they are regarded as “superfoods” due to their high protein, vitamin, and mineral content [58, 61]. Other moderately utilized species, such as *Carissa edulis*, *Grewia mollis*, *Ficus vasta*, and *Vachellia seyal*, provide both food and secondary benefits including fodder and minor income, demonstrating the multifunctionality of WEPs in sustaining rural livelihoods.

Conversely, species like *Datura stramonium*, *Lantana camara*, and *Solanum nigrum* were less frequently used due to toxicity concerns or limited palatability. This reflects a deliberate risk-averse strategy by local communities, emphasizing indigenous knowledge in differentiating safe from harmful species. Similar avoidance patterns have been documented globally; for example, in Europe and North America, toxic or low-palatable wild plants are traditionally excluded from routine diets despite local availability [92].

### Newly identified wild edible plants in Karamara forest patches

The documentation of newly identified WEPs in the Karamara forest patches reveals the underexplored botanical diversity of the Somali Region and expands the regional ethnobotanical inventory. Among the 42 recorded species, ten were reported for the first time in this locality, including *Amaranthus spinosus*, *Cucumis kelleri*, *Ehretia cymosa*, *Senna occidentalis*, *Acacia mellifera, Senna occidentalis, Tamarindus indica, Canthium coromandelicum*. These findings demonstrate that even well-known forest patches can harbor previously undocumented species, emphasizing the importance of site-specific ethnobotanical surveys for capturing local biodiversity and traditional knowledge [3, 21].

Several of the newly recorded species serve as either additional foods (AF) or famine foods (Fam), with edible parts including fruits, leaves, seeds, or whole plants. For instance, *Amaranthus spinosus* contributes leafy greens and seeds, while *Cucumis kelleri* provides a seasonal fruit resource. This dual categorization underlines their potential role in enhancing dietary diversity and resilience, particularly in dryland and food-insecure regions. Similar patterns are observed in Eastern Ethiopia and other African countries, where newly documented or underutilized wild plants often serve as supplementary or emergency food sources during lean seasons [13, 52, 60].

The occurrence of species such as *Amaranthus spinosus* and *Cucumis kelleri* in the wild, despite their cultivation elsewhere, highlights the complex interplay between wild and semi-cultivated food systems. In South Asia, for example, several *Amaranthus* species exist both as wild and domesticated forms, reflecting local management practices and selective harvesting [61]. Similarly, *Senna occidentalis*, and *Tamarindus indica* are known for their fruits in other African regions but had not been previously recorded as part of the Somali Region’s ethnobotanical repertoire [90, 91, 95].

The documentation of these species contributes to global ethnobotanical knowledge, offering comparative insights with regions in Africa, Asia, and beyond, where WEPs remain crucial for food security and cultural identity [59, 62, 64, 95].

### Implications of using wild edible plants in Karamara forest patches

The WEPs of the Karamara forest patches perform critical roles in sustaining local food security, nutrition, cultural practices, and livelihoods. Leafy vegetables such as *Amaranthus caudatus*, *A. dubius*, *A. spinosus*, along with *Opuntia ficus-indica* and *Commelina latifolia*, enhance dietary diversity by supplementing grain-based diets, particularly during lean seasons. These findings are consistent with other Ethiopian ethnobotanical studies, where *Amaranthus* species and drought-tolerant herbs provide essential micronutrients and serve as key dietary supplements during periods of scarcity [21, 32, 41, 50]. Comparable observations exist in South Asia, Africa, and Latin America, where WEPs function as nutritional buffers in rural diets, especially in semi-arid and food-insecure regions [26, 59, 62, 92].

Famine foods, including *Rhus vulgaris*, *Carissa edulis*, *Cordia africana*, and *Cucumis kelleri*, act as critical safety nets during droughts or crop failures, reflecting the resilience of traditional food systems. Similar reliance on famine species has been reported in the Sahel, Tanzania, and parts of India, highlighting a universal strategy of risk mitigation through underutilized WEPs [60, 61, 90]. This dual classification of additional and famine foods highlights the nuanced local knowledge that informs the timing, selection, and preparation of wild resources.

Beyond nutrition, several WEPs possess bioactive compounds with ethnomedicinal value. *Moringa stenopetala*, *Ziziphus mauritiana*, and *Ricinus communis* are traditionally used to prevent nutrient deficiencies and treat minor ailments, consistent with reports from Ethiopia, Pakistan, and India that highlight their antioxidant, antimicrobial, and anti-inflammatory properties [24, 64, 72, 76]. The combined nutritional and medicinal functions emphasize the integrated role of WEPs in enhancing human health and supporting local healthcare practices in resource-limited settings.

Certain species also contribute to household income, particularly *Balanites aegyptiaca*, *Ziziphus mucronata*, and *Moringa stenopetala*, which are marketable due to their fruits and leaves. This reflects patterns seen in Ethiopia and throughout Africa, where WEPs with market value support rural livelihoods and strengthen economic resilience [57, 95].

The multi-purpose nature of these species serving simultaneously as food, medicine, fuel, fodder, or construction material further underlines their critical role in sustaining community well-being and coping with environmental variability.

From an ecological perspective, WEPs such as *Cordia africana*, *Ficus sycomorus*, and *Acacia tortilis* contribute to habitat structure, biodiversity maintenance, and the provision of ecosystem services while supplying edible products. However, pressures including deforestation, overgrazing, and invasive species threaten the sustainability of these resources, highlighting the urgent need for community-based management, conservation strategies, and sustainable harvesting protocols. Globally, studies from Africa, Asia, and Latin America similarly emphasize that the long-term viability of WEPs depends on integrating ecological stewardship with local knowledge systems and livelihood strategies [63, 67].

### Priority ranking of factors affecting wild edible plants

The priority ranking of factors affecting the availability and sustainability of WEPs in the Karamara forest patches highlights the complex interplay of anthropogenic pressures and environmental constraints shaping local plant resources. Firewood collection emerged as the foremost threat, reflecting the high dependence of rural households on forest biomass for domestic energy. Similar patterns have been reported across Ethiopia, including in South Gondar and Soro districts, where unsustainable harvesting for fuelwood directly reduces the availability of multipurpose WEPs and compromises forest regeneration [55, 57]. Globally, reliance on forest products for household energy is recognized as a leading driver of habitat degradation, particularly in semi-arid and tropical dryland ecosystems of Africa, Asia, and Latin America [85, 87, 91, 92].

Charcoal production ranked second in priority, reflecting its growing role as a cash-generating activity. The extraction of woody biomass for charcoal disproportionately affects multipurpose trees such as *Ficus sycomorus*, *Cordia africana*, and *Acacia spp.*, which provide fruits, fodder, and medicinal resources. Comparable findings from West Africa, India, and Pakistan demonstrate that charcoal production often accelerates deforestation and threatens the sustainability of wild food resources [61, 76, 90, 91]. This emphasizes the need for integrated forest management strategies that balance local livelihoods with the conservation of critical WEP species.

Overgrazing was ranked third, highlighting the impact of livestock pressure on natural regeneration and habitat integrity. This threat is particularly pronounced in dryland ecosystems, where heavy grazing suppresses seedling establishment and alters plant community composition, as observed in the Sahel, miombo woodlands of Tanzania, and other Ethiopian rangelands [21, 30, 60]. Similarly, recurrent drought, ranked fourth, highlights the vulnerability of WEPs to climate variability. Seasonal and interannual fluctuations in precipitation affect fruiting and leaf availability, influencing both dietary access and ecosystem resilience. Comparable studies in Ethiopia and Asia report that drought periods exacerbate food insecurity and reduce the abundance of key wild plants [50, 62].

Other identified threats, including agricultural expansion, invasive alien species, and the use of plants for construction, further intensify pressure on WEPs. Agricultural encroachment converts natural habitats, while invasive species compete with native plants, reducing species richness and availability for local communities. These pressures align with global observations, where land-use change and invasive flora are among the primary challenges to sustaining wild food plant populations in Africa, Asia, and Europe [61, 68, 72, 92].

Systematic monitoring and policy interventions are essential to preserve the ecological, nutritional, and cultural value of WEPs in Karamara, consistent with best practices observed in Ethiopia and comparable dryland regions globally [50, 68].

### Conservation and management of wild edible plants in Karamara forest patches

The Karamara forest patches harbor a rich diversity of WEPs that are essential to local food security, nutrition, livelihoods, and traditional medicine. However, the sustainability of these resources is increasingly threatened by multiple anthropogenic and ecological pressures, including firewood collection, charcoal production, overgrazing, agricultural expansion, invasive species such as *Prosopis juliflora*, and recurrent droughts. These pressures not only reduce the availability of WEPs but also disrupt traditional knowledge transfer, which is critical for the continued use and management of these species [2, 3, 13, 21]. Similar challenges have been documented across Africa, Asia, and Latin America, highlighting a widespread concern for the conservation of wild food plants under increasing human and climatic pressures [64, 67, 91].

The diversity of WEPs in terms of life forms, edible parts, and collection methods highlights the need for tailored management strategies. Herbs and leafy vegetables, including *Amaranthus spp.*, *Commelina latifolia*, and *Solanum nigrum*, are predominantly harvested by plucking, while shrubs and trees such as *Ficus sycomorus*, *Ziziphus spp.*, and *Cordia africana* are mainly collected for fruits and gums through picking. These methods have different implications for sustainability: while plucking can be labor-intensive and occasionally damaging if repeated excessively, picking is generally less destructive and supports regenerative cycles [59, 63]. Recognizing these species-specific harvesting dynamics is essential for designing conservation interventions that minimize ecological impacts while maintaining community access.

The integration of cultivated or semi-cultivated species, such as *Moringa stenopetala* and *Opuntia ficus-indica*, into homegardens represents an effective ex-situ conservation strategy. This approach not only ensures year-round availability of key WEPs but also reduces pressure on wild populations, aligning with agroforestry and sustainable land-use models reported in Ethiopia, India, and China [50, 61, 64]. Moreover, these species provide multiple benefits, including nutritional supplementation, income through marketable products, and medicinal applications, reinforcing their socio-economic importance [32, 48, 57].

Community-based management emerges as a central pillar for WEP conservation in Karamara. Local households rely on WEPs for supplementary foods during lean periods, marketable species for income generation (e.g., *Opuntia ficus-indica*, *Tamarindus indica*, *Balanites aegyptiaca*), and medicinal plants for primary healthcare. Participatory approaches that combine indigenous knowledge with formal conservation planning have been successful in similar dryland and tropical ecosystems, ensuring sustainable harvesting, habitat protection, and knowledge transmission across generations [30, 67, 68, 72].

Furthermore, the management of invasive species, regulation of unsustainable fuelwood and charcoal collection, and promotion of alternative livelihoods are essential to maintain both ecological and social resilience. Integrating WEP conservation into broader landscape management strategies can help balance community needs with ecosystem sustainability, providing a model for other regions in Ethiopia, Africa, and globally where WEPs play a critical role in food security, nutrition, and cultural heritage [2, 51, 92].

### IUCN red list status of wild edible plants

The conservation status of WEPs in the Karamara forest patches remains largely undocumented, presenting a challenge for sustainable management and prioritization. Preliminary assessments indicate that while some species such as *Moringa stenopetala*, *Cordia africana*, *Tamarindus indica*, and *Balanites aegyptiaca* are classified as Least Concern (LC) by the International Union for Conservation of Nature (IUCN), the majority of species have not been evaluated [98]. This gap emphasizes the need for comprehensive, site-specific assessments to determine population trends, threats, and conservation priorities, particularly for species heavily utilized for food, medicine, and livelihood purposes.

Globally, under-documentation of WEPs is a common challenge, especially in tropical and subtropical regions, where ethnobotanical knowledge exists but formal conservation assessments lag behind [64, 68]. In Ethiopia, similar patterns have been observed; studies in the Somali, Amhara, and Tigray regions report numerous locally important WEPs that remain unassessed by the IUCN, despite their critical roles in nutrition, income, and ecosystem services [2, 21, 40]. This lack of formal conservation status can result in the inadvertent overharvesting of vulnerable species and inadequate attention in management and policy frameworks.

The preliminary LC status of some key species, such as *Moringa stenopetala* and *Cordia africana*, provides reassurance regarding their current population stability. However, this classification does not capture localized pressures, such as firewood collection, overgrazing, and invasive species, which may lead to rapid declines in specific forest patches [57, 59]. Therefore, a combination of formal IUCN assessment and local ecological monitoring is essential to identify threatened species early and implement targeted conservation strategies.

Cultivated or semi-cultivated WEPs, such as *Moringa stenopetala* and *Opuntia ficus-indica*, can be integrated into homegardens to reduce pressure on wild populations, while multipurpose trees like *Cordia africana* can benefit from community-based protection measures and sustainable harvesting protocols [61, 63]. Moreover, raising awareness among local communities regarding species at risk, even if globally categorized as Least Concern, can enhance stewardship and ensure that culturally important WEPs remain available for future generations.

### Wild edible plant knowledge differences among informant groups

The study revealed significant variation in wild edible plant (WEP) knowledge among informant groups in the Karamara forest patches, reflecting the influence of gender, literacy, age, and local expertise on ethnobotanical knowledge. Male informants reported a higher mean number of WEPs than women, a pattern consistent with findings in Ethiopia and other countries where men often engage more in forest-based activities such as herding, hunting, and wood collection, which increases exposure to diverse plant species [2, 3, 21, 59]. Women, while actively involved in household food preparation, may rely more on cultivated or locally available species, which can reduce their breadth of WEP knowledge relative to men.

Literacy status also significantly affected WEP knowledge, with illiterate participants demonstrating higher familiarity with wild plant species than literate individuals. This aligns with studies from Ethiopia, Pakistan, and India, where illiterate or less formally educated individuals often possess greater traditional ecological knowledge due to their deeper reliance on local natural resources for subsistence and household livelihoods [13, 57, 61]. Literate individuals may increasingly depend on market foods and modern agricultural products, resulting in reduced engagement with wild resources.

Experience and social recognition were particularly important determinants of WEP knowledge. Key informants, recognized as local experts, consistently reported higher numbers of species than general informants. This validates the purposive selection of key informants in ethnobotanical studies, highlighting their role as custodians of traditional knowledge and as transmitters of culturally significant practices [64, 68]. Similarly, elder informants possessed significantly more knowledge than middle-aged and young participants, emphasizing the accumulation of experiential knowledge over a lifetime and the importance of elders in intergenerational knowledge transfer. The strong positive correlation between age and WEP knowledge (R = 0.66, p < 0.001) further reinforces this trend, echoing findings from other Ethiopian regions and across Africa and Asia, where youth often show declining engagement with wild plants due to modernization and shifts toward market-oriented diets [3, 32, 40, 92].

Ethnobotanical education programs and community-based knowledge transmission initiatives can help bridge this gap and ensure that traditional knowledge continues to support food security, nutrition, and sustainable management of WEPs [2, 50, 57, 59]. Recognizing gendered differences in knowledge can inform targeted conservation and training interventions, ensuring that both men and women participate in sustainable harvesting practices and the protection of culturally and nutritionally important species. The observed influence of literacy and experience suggests that integrating local ecological knowledge with formal education and participatory management strategies can strengthen both conservation outcomes and community resilience in the face of environmental and socio-economic change.

### Indigenous knowledge transfer and practices of wild edible plants

The findings indicate that the use of WEPs in the Karamara forest patches is deeply embedded in indigenous knowledge systems that regulate species identification, harvesting, preparation, and consumption. Knowledge acquisition is predominantly oral, transmitted through observation, guidance from elders, storytelling, and participation in communal practices. This pattern aligns with studies in Ethiopia and other countries, where intergenerational knowledge transfer is the main pathway for preserving ethnobotanical knowledge and ensuring safe and effective use of wild resources [55, 64, 91].

Direct observation and experiential learning were reported by the majority of informants, reflecting the hands-on nature of ethnobotanical knowledge acquisition. Learning through elders, oral histories, and cultural narratives reinforces not only the technical aspects of plant use but also the ethical and cultural values associated with WEPs. Similar mechanisms have been observed in rural India, Pakistan, and across sub-Saharan Africa, where elders and key informants act as custodians of ecological knowledge, teaching younger generations to distinguish edible species, avoid toxic plants, and respect seasonal harvesting rules [40, 76, 85, 95].

Traditional practices in Karamara include sustainable harvesting methods tailored to plant life forms and growth habits. Selective plucking of leaves, careful picking of fruits, and controlled digging of roots ensure minimal damage and promote regeneration. These techniques are consistent with community-based conservation practices documented in other dryland regions of Ethiopia and lowland Africa, where sustainable harvesting rules are integral to maintaining both food security and biodiversity [48, 54, 91]. The focus on famine foods, such as *Solanum nigrum*, *Rubus volkensii*, and *Senna occidentalis*, highlights the role of indigenous knowledge in preparing emergency resources safely, ensuring households have reliable food during droughts or crop failures.

Preparation methods in Karamara demonstrate sophisticated knowledge aimed at improving palatability and reducing toxicity. Boiling, roasting, or processing specific plant parts such as tubers of *Ricinus communis* or resins from *Acacia tortilis* and *Vachellia seyal* reflects practical applications of chemical and sensory understanding acquired over generations. Similar ethnobotanical practices have been reported globally, including in China, India, and Latin America, where local processing methods enhance the safety, flavor, and nutritional value of wild foods [61, 63, 68, 95].

## Limitations of the study

This study was constrained by its relatively small sample size and focus on selected forest patches, which may not fully represent the diversity of WEPs across the wider Karamara landscape. Although only 64 informants were surveyed, the findings provide valuable insights into local indigenous knowledge of WEPs. Seasonal limitations restricted observations of certain species’ phenology and availability.

## Conclusion and recommendations

The ethnobotanical survey of the Karamara forest patches in Eastern Ethiopia documented 42 wild edible plant (WEP) species, highlighting their critical roles in local food security, nutrition, health, and livelihoods. Shrubs and trees dominated the species composition, with fruits and leaves being the most commonly consumed parts. WEPs function both as supplementary foods and as vital famine resources, underscoring their importance in seasonal and emergency dietary strategies. Indigenous knowledge is central to the sustainable use of these plants, encompassing species identification, harvesting techniques, preparation methods, and safe consumption practices. Knowledge transmission occurs primarily through oral tradition, observation, and hands-on experience, with elders and key informants serving as principal custodians. Quantitative analyses revealed significant variations in WEP knowledge across gender, age, literacy level, and experience, highlighting the need to engage knowledgeable community members in conservation and educational initiatives. The study also confirmed the multifunctional value of WEPs, which provide food, medicine, fodder, fuelwood, construction materials, and marketable products. Preference and ranking exercises identified highly valued species, including *Amaranthus* spp., *Moringa stenopetala*, *Ficus sycomorus*, *Ziziphus mauritiana*, and *Balanites aegyptiaca*, while species with potential toxicity, such as *Datura stramonium* and *Lantana camara*, are used cautiously. Seasonal availability and adaptive collection strategies demonstrate local efforts to optimize access while minimizing ecological impacts.

However, anthropogenic pressures including firewood collection, charcoal production, overgrazing, agricultural expansion, and invasive species pose significant threats to WEP sustainability. To address these challenges, immediate conservation and sustainable management strategies are needed through collaboration among local authorities, community members, and conservation organizations. Awareness campaigns and capacity-building initiatives should target all community members to promote sustainable use and highlight the medicinal and nutritional potential of WEPs. Integrating indigenous knowledge into formal education and extension programs can strengthen community engagement and intergenerational knowledge transfer.

Future research should focus on the nutritional composition, phytochemistry, and pharmacological properties of WEPs to ensure their safe use and potential wider applications. Enhancing local market infrastructure and value chains for WEP products can generate income particularly for women and marginalized groups while incentivizing conservation through sustainable commercialization. Finally, recognizing the significance of WEPs in regional and national policies will support holistic rural development and biodiversity conservation, ensuring these vital plant resources continue to benefit present and future generations.

## Data Availability

No datasets were generated or analysed during the current study.

## Abbreviations

ANOVA: Analysis of variance
CSA: Central statistical agency of Ethiopia
IK: Indigenous knowledge
MTU: Mizan-Tepi University
WEP: Wild and semi wild edible plants
BEI: Botanical ethnoknowledge index
IPNI: International plant names index
POWO: Plants of the world online
